# Exploring Guanidinium Group Involvement in Hordatine Interactions with the G-Quadruplex Motif Within the c-MYC Promoter Region

**DOI:** 10.3390/ijms262110580

**Published:** 2025-10-30

**Authors:** Denise Dozio, Aziza Caccia, Sabrina Dallavalle, Giovanni Luca Beretta, Paola Perego, Roberto Artali, Stefania Mazzini, Salvatore Princiotto

**Affiliations:** 1 Department of Food, Environmental and Nutritional Sciences (DeFENS), Università degli Studi di Milano, Via Celoria 2, 20133 Milan, Italy; denise.dozio@unimi.it (D.D.); aziza.caccia@unimi.it (A.C.); sabrina.dallavalle@unimi.it (S.D.); salvatore.princiotto@unimi.it (S.P.); 2 Molecular Pharmacology Unit, Department of Experimental Oncology, Fondazione IRCCS Istituto Nazionale Tumori, Via Amadeo 42, 20133 Milan, Italy; giovanni.beretta@istitutotumori.mi.it (G.L.B.); paola.perego@istitutotumori.mi.it (P.P.); 3 Scientia Advice di Roberto Artali, Via Garibaldi 58, 20811 Cesano Maderno, Italy; roberto.artali@scientia-advice.com

**Keywords:** G-quadruplex, guanidinium, hordatine, NMR spectroscopy, docking, cytotoxic activity

## Abstract

G-quadruplexes (G4s) are four-stranded DNA or RNA structures formed by guanine-rich sequences. They occur in functional regions of the genomic material, including the promoter part of genes, regulatory region, and telomeric threads. G4s play a key role in various biological processes, including transcription, replication, and telomere maintenance. Guanidine-containing derivatives can bind to G-quadruplexes, either by intercalating into the structure or by interacting with the grooves or loops. The binding can stabilize the G-quadruplex, potentially affecting its biological function. In this paper, the ability of guanidinium-containing hordatines to interact with G4 was evaluated. Analogues lacking the guanidinium group or showing the benzofuran system instead of the dihydrobenzofuran core were prepared and tested as well. NMR titration and docking calculations were used to probe the binding of the compounds to G4 of *c-MYC* oncogene. Spectroscopic analyses were consistent with a significant interaction of benzofurans **3** and **4** at the 5′-end and 3′-end tetrads and with the formation of ligand/G-quadruplex complexes with a 2:1 stoichiometry. The resulting data were supported by docking simulations. Cytotoxic activity was evaluated on a model of U2OS osteosarcoma (ATCC HTB-96) and breast cancer (MDA-MB-231) cell lines, further highlighting the key role of the guanidinium fragment and the benzofuran core in the G-quadruplex stabilization.

## 1. Introduction

The interaction of small molecular ligands with DNA is a pivotal area of research in the development of novel cancer therapeutics [1,2]. However, traditional DNA-targeting agents that bind double-stranded DNA often suffer from poor sequence selectivity, leading to off-target effects and significant toxicity [3]. To address this limitation, alternative DNA structures with non-canonical conformations have emerged as more selective therapeutic targets.

Nucleic acids are highly dynamic molecules capable of adopting a variety of secondary and tertiary structures, depending on their sequence and cellular microenvironment. Among these, G-quadruplexes (G4s)—four-stranded DNA structures formed by guanine-rich sequences—have garnered significant attention [4,5]. Under physiological conditions, certain single-stranded guanine-rich regions can fold into G4 structures, stabilized by G-quartets, which are planar arrangements of four guanine bases held together by Hoogsteen hydrogen bonds.

These G-quartets stack on top of one another to form the G-quadruplex architecture. The stability of these structures is further enhanced by the presence of monovalent cations, particularly potassium (K^+^) and sodium (Na^+^), which are physiologically abundant and promote the stacking of G-quartets into stable G4 assemblies.

G4 structures are not randomly distributed throughout the genome; rather, they are enriched in specific genomic regions such as the promoter regions of proto-oncogenes (*BCL-2*, *KRAS*, *c-KIT*, *c-MYC*), telomeric ends, and transcription factor binding sites [6]. The presence of G4 motifs in these regulatory regions has been linked to both genetic instability and altered gene expression, contributing to tumorigenesis. Consequently, the selective stabilization of G-quadruplex structures by small-molecule ligands has emerged as a promising therapeutic strategy to suppress the transcription of oncogenes and potentially inhibit cancer progression [7].

Among the key oncogenes implicated in cancer is *c-MYC*, which encodes a transcription factor involved in regulating crucial cellular processes such as cell-cycle progression, differentiation, apoptosis, DNA replication, and mRNA maturation. Under physiological conditions, *c-MYC* expression is tightly controlled. However, in many tumor types, *c-MYC* is frequently amplified or dysregulated, contributing significantly to oncogenesis.

The transcriptional regulation of *c-MYC* is complex and involves multiple regulatory elements and DNA-binding proteins. A critical region involved in its regulation is a GC-rich sequence known as *Pu27*, located in the promoter region. This sequence can fold into intramolecular G-quadruplex (G4) structures, which have been shown to repress *c-MYC* transcription when stabilized [8,9].

Given that *c-MYC* overexpression is one of the most common molecular abnormalities across a wide spectrum of human cancers, the stabilization of G-quadruplex structures within its promoter by small molecules has emerged as a promising antitumor strategy [10].

Over the past few decades, a wide range of G-quadruplex (G4) ligands with diverse chemotypes and structural features have been explored as potential *c-MYC* promoter-binding molecules [11,12,13]. These compounds typically share a common architecture: a polyaromatic core that enables π–π stacking interactions with the G-quartets, complemented by positively charged side chains that facilitate binding to the DNA phosphate backbone and interactions with water molecules within the grooves.

Our research group has long been dedicated to the discovery and characterization of novel chemotypes targeting the G4 structure within the *c-MYC* promoter. In recent years, our focus has shifted toward natural products, particularly those derived from plants [14,15,16,17], and previously studied in the context of G4 interactions. Specifically, we investigated the G4-stabilizing potential of plant-derived stilbenoids, including dimeric derivatives of resveratrol and pterostilbene-namely, δ-viniferin and pterostilbene-*trans*-dihydrodimer. These compounds feature a dihydrofuran core linked to hydroxyl- and methoxy-substituted aromatic rings, offering a structural motif compatible with G4 binding.

Building on these findings, we hypothesized that G4 interaction could be further enhanced by introducing positively charged side chains onto these natural scaffolds. These moieties are known to play a critical role in stabilizing G4 structures through electrostatic interactions with the negatively charged DNA backbone, as described above.

In this context, we became particularly interested in a distinct class of dimeric natural compounds containing a dihydrofuran core, namely, the hordatines. Hordatines are phenolic secondary metabolites characteristic of barley and are formed through the dimerization of hydroxycinnamic acid agmatines, such as *p*-coumaroylagmatine and feruloylagmatine. Specifically, hordatine A is a dimer of *p*-coumaroylagmatine, hordatine B is a heterodimer of feruloylagmatine and *p*-coumaroylagmatine, and hordatine C is a dimer of feruloylagmatine [18,19].

Our interest in these compounds was sparked by the presence of the guanidinium group in their structure, which could potentially enhance G-quadruplex (G4) stabilization through electrostatic interactions with the phosphate backbone of DNA. There is growing evidence that guanidine-containing derivatives can selectively bind and stabilize G-quadruplex structures [20]. For instance, guanidinium-functionalized compounds have been shown to target the G-quadruplex within the promoter region of the *BCL-2* gene, a key regulator in cancer development [21]. Stabilization of this G4 structure has been associated with transcriptional repression of *BCL-2*, thereby potentially inhibiting cancer cell proliferation.

Moreover, several guanidine derivatives have demonstrated cytotoxic activity against cancer cells, further supporting their potential as anticancer agents [22]. The guanidinium group not only facilitates DNA binding but also contributes to enhanced water solubility and favorable hydrophobic interactions, aiding drug delivery into tumor cells while potentially reducing systemic toxicity [23].

Based on these considerations, we set out to evaluate the ability of the natural compound **1** to stabilize the G-quadruplex structure located in the promoter region of the *c-MYC* oncogene.

**Figure 1:**
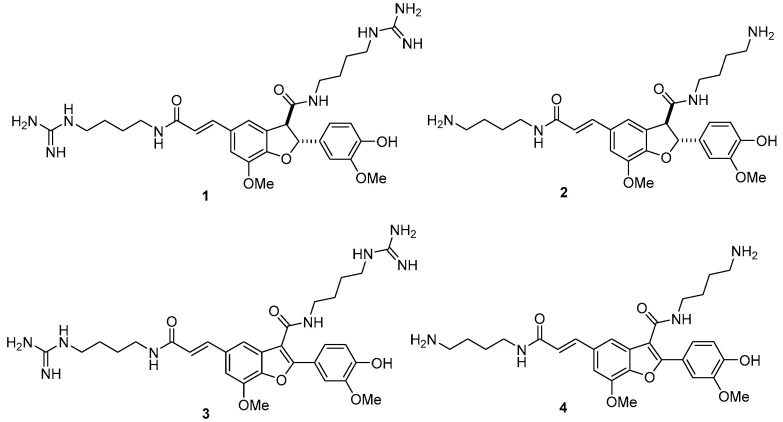
Structure of compounds **1**–**4**.

To evaluate the specific contribution of the guanidinium group to G-quadruplex binding, we synthesized a derivative (compound **2**) that retains the molecular scaffold and positively charged side chains of compound **1**, with identical carbon chain lengths, but replaces the guanidinium moieties with amino groups (Figure 1). This modification allows us to directly compare the contribution of the guanidinium functionality to G4 stabilization.

In addition, we prepared analogues of compounds **1** and **2** in which the dihydrobenzofuran core was replaced by a benzofuran core (compounds **3** and **4**, respectively, Figure 1). This structural variation was designed to evaluate the effect of molecular geometry on G-quadruplex interaction under the rationale that a planar aromatic system may enhance π–π stacking with the G-quartets and thereby improve binding affinity and stabilization.

## 2. Results and Discussion

**Scheme 1:**
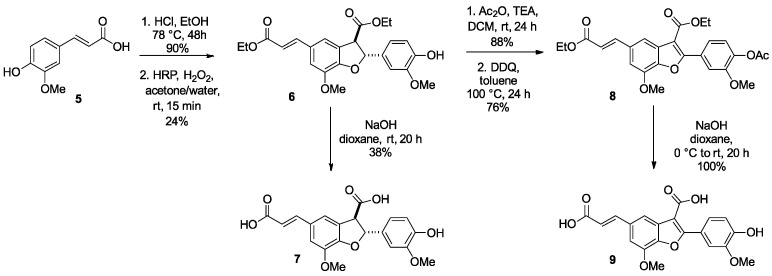
Synthesis of acids **7** and **9**.

**Scheme 2:**
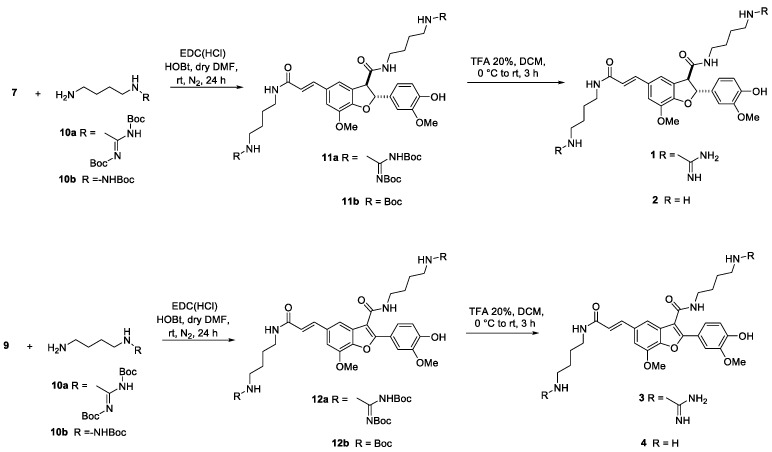
Synthesis of compounds **1–4**.

Compound **1** was prepared starting from ferulic acid **5**, as described in Scheme 1 [24]. The acid was converted into the corresponding ethyl ester, which was treated with HRP/H_2_O_2_ in acetone/water to give dimer **6** as a racemic mixture. Basic hydrolysis with 2 M NaOH afforded the acid **7**. The acid **9** was obtained from the same intermediate **6** by acylation with Ac_2_O followed by a DDQ-mediated oxidation to give the ester **8**. Basic hydrolysis afforded compound **9** in quantitative yield. Amide coupling of **7** with bis Boc-protected agmatine **10a** and mono Boc-protected putrescine **10b**, in the presence of EDC HCl and HOBt as coupling agents, followed by deprotection with TFA, gave compounds **1** and **2**, respectively. Compounds **3** and **4** were synthesized analogously, starting from dimer **9**. (Scheme 2).

### 2.1. NMR and Docking Binding Studies

To study the interactions of compounds **1**–**4** with G-quadruplex, NMR titration experiments for all the compounds with Pu22T14T23 were performed. Chemical shift variations and the broadening of the imino protons from the G-quadruplex located at 10−12 ppm in the NMR spectrum upon the addition of increasing compound concentrations were monitored.

#### 2.1.1. NMR Experiments

NMR spectroscopy was used to probe the binding of compounds **1**–**4** to G4 of c-MYC oncogene. The purine-rich strand of the nuclease hypersensitive element (NHE) III sequence of the c-MYC promoter, which controls 80–90% of the c-MYC transcription is a 27 nt segment (MYCPu27), containing five consecutive runs of guanines. Pu22 is a 22-mer sequence of MYCPu27, mainly responsible for the c-MYC transcriptional activity. Specifically, the experiments were performed with the sequence Pu22T14T23, which contains two mutations at positions 14 and 23 of c-MYC Pu22 with respect to the native sequence [25]. This modified oligonucleotide has been used by several groups to study the binding properties of G-quadruplex binding drugs by NMR, as the wild sequence gave poorly resolved proton NMR spectra. Pu22T14T23 forms a single monomeric intramolecular parallel G-quadruplex structure like the native form. Under our experimental conditions, the sequence forms a single G4 conformation characterized by 12 well-resolved imino proton peaks, corresponding to the 12 guanines involved in the three G-tetrad planes: i.e., G7-G11-G16-G20, G8-G12-G17-G21, and G9-G13-G18-G22, each comprising four Hoogsteen-type hydrogen-bonded guanine residues.

**Figure 2:**
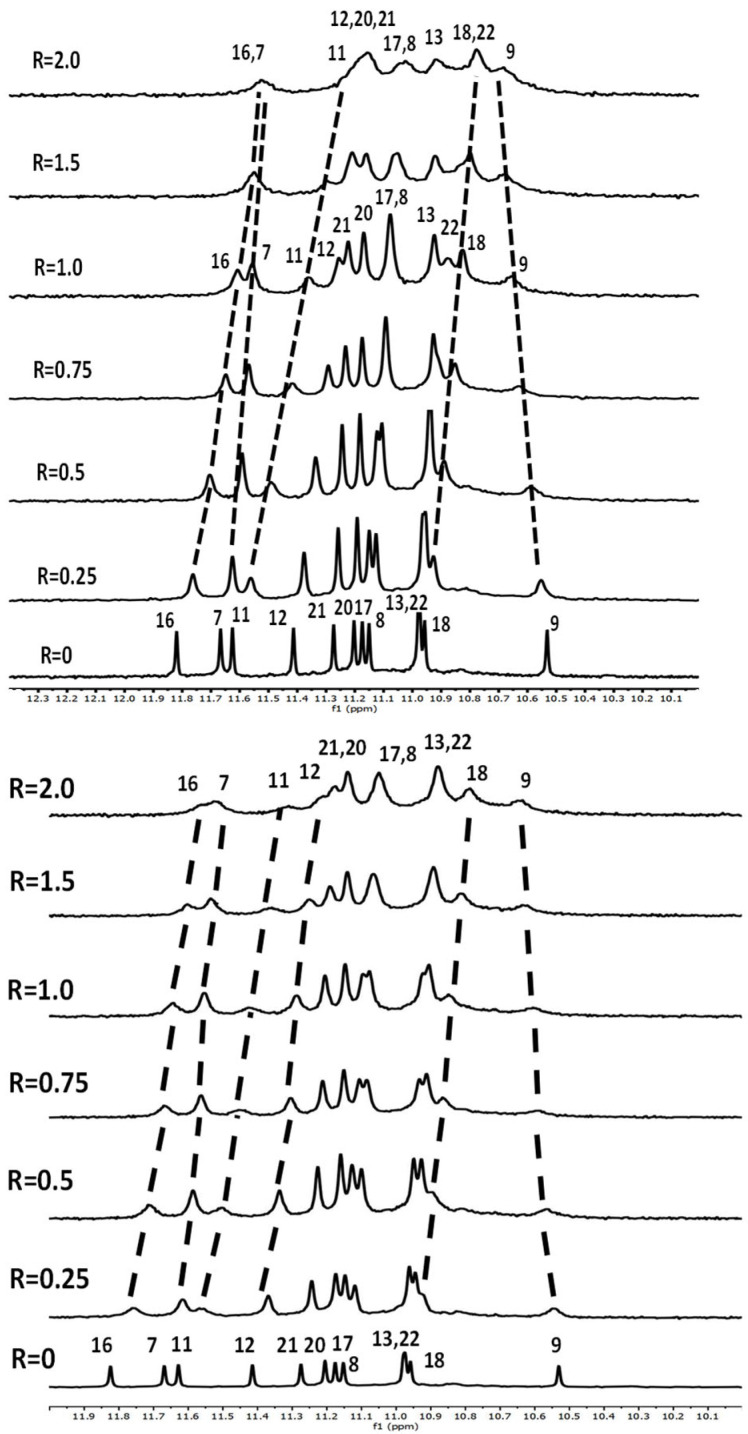
Imino proton region of the 1D NMR titration spectra of Pu22T14T23 with **4** (**top**) and with **3** (**bottom**) at 25 °C in H_2_O/D_2_O (9:1), 25 mM KH_2_PO_4_, 70 mM KCl, pH 6.9, at different R = [ligand]/[DNA] ratios.

Imino protons of the Pu22T14T23 G-tetrads in the complexes were assigned by tracking in the 1D titration spectra upon the addition of ligands (Figure 2, Figures S1 and S2) and validated by inter-residue NOE interactions. Information concerning the binding interactions was deduced by observing the impact on the imino protons in the 5′-end and 3′-end quartets: large changes in chemical shift were observed for some G-quadruplex imino proton signals upon the addition of increasing amounts of **3** and **4** (Figure 2, Tables S1 and S2). Less marked or no significant effects on NMR signals were detected upon the addition of **1** and **2** (Figures S1 and S2 and Table S3).

For compounds **3** and **4**, a generalized line broadening occurred in the titration experiments, indicating that the ligand binding process was at an intermediate rate relative to the NMR time-scale and that, probably, various binding events occurred (Figure 2). The largest shifts variations were observed for the imino proton signals of G-tetrads belonging to the 5′-(G16, G11 and G7) and 3′-end (G18 and G22). These findings are consistent with π stacking of **3** and **4** at the 5′-end and 3′-end tetrads and with the formation of ligand/G-quadruplex complexes with a 2:1 stoichiometry. Significant line broadening was also observed in the aromatic proton regions (Figure S3).

To further explore the nature of the G-quadruplex-**3** and G-quadruplex-**4** interactions, NOESY and TOCSY experiments were conducted.

#### 2.1.2. Interaction of Pu22T14T23 with **3**

**Figure 3:**
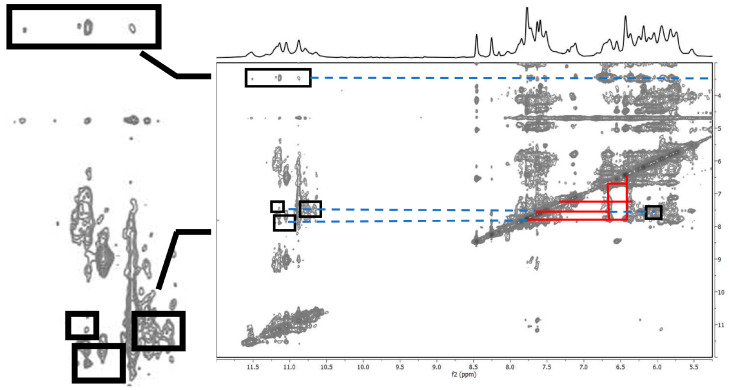
Imino and aromatic protons region of the 2D-NOESY spectrum at 25 °C in H_2_O/D_2_O (9:1), 25 mM KH_2_PO_4_, 70 mM KCl, pH 6.9, at R = [3]/[DNA] = 2.0. The boxes and the blue dashed lines show the NOE interactions between imino protons of Pu22T14T23 and aromatic protons and OCH_3_ of compound **3**. The red line indicates the intramolecular NOE interactions between aromatic protons of **3**.

The NH imino protons were used to assign the G-tetrad base aromatic protons (Tables S1 and S2). However, only A15H8, A15H2, T23 and the groups T14, A6, and G22 could be assigned (Figure S3). The remaining guanine assignments were determined by analyzing inter-residue NOE interactions with the imino NH protons, which reconstructs the patterns of the three tetrads, i.e., G7NH–G11H8, G11NH–G16H8, G16NH–G20H8, G20NH–G7H8 (tetrad I); G8NH–G12H8, G12NH–G17H8, G17NH–G21H8, G21NH–G8H8 (tetrad II); G9NH–G13H8, G13NH–G18H8, G18NH–G22H8, G22NH–G9H8 (tetrad III) (Figure S4). The assignment of the thymine H6 and CH_3_ protons followed the 1D titration spectra, because their shifts remained quite constant. CH_3_ signals were sharp, except for T23 resonance that, although very broad, was detected at 1.66 ppm. Aromatic protons of compound **3** in the complexed state were evidenced. Consequently, although only a few intermolecular NOE signals were detected, their significance provided clear evidence of direct interactions involving the G-tetrads located at both the 5′ and 3′ ends of the structure (Figure 3 and Table 1).

Specifically, NOE interactions at the level of the 3′-end connect G18 and G22 residues to an aromatic proton and OCH_3_ group, thus confirming the placement at the top of the external tetrad. At the 5′-end, the NOE contacts involving G7 and G20 were detected. This is in line with the significant up-field shift of the imino protons of the guanines G7, G11, and G16, because of the presence of a second molecule of compound **3** located at the top of the tetrad. All the intermolecular NOE are reported in Table 1.

#### 2.1.3. Interaction of Pu22T14T23 with **4**

The imino proton signals appeared broad and overlapped, thus precluding an accurate analysis. For the complex Pu22T14T23/**4**, NOE intermolecular interactions between the OCH_3_ groups and NH imino protons belonging to both tetrads were observed: OCH_3_ group showed interactions with G16, G9, G12, G13, G18, G21, and G22 (Figure S5).

### 2.2. Docking Studies

To visualize the potential binding mode of **3** and **4** docking calculations for the c-Myc gene promoter were performed at both 5′- and 3′-ends of the parallel G4 structure (PDB access code: 2L7V). This approach was guided by our experimental findings, which showed that **3** and **4** bind to the target DNA in a 2:1 ratio, interacting with both terminal regions. In this context, it was chosen to use the solvation energy term values of the optimized complexes to compare the influence of the different solvent exposure of the compounds in the two possible 3′-end and 5′-end conformations.

#### 2.2.1. Docking Analysis of the Interaction Between Pu22T14T23 and **3**

The most frequent docking poses of **3** at the 3′-end and 5′-end binding sites are consistent with the NMR data, which show chemical shift perturbations in the imino protons of these tetrads in the presence of **3**. The docked ligand forms mainly cation–π, π–π, ionic interactions and H-bonds. Specifically, at 3′-end binding site **3** is not centered with the tetrad but stacks with G18 and forms an H-bond with OH of phenol moiety. One side chain protrudes towards G13 and forms, via amide group, a hydrogen bond with this residue. Moreover, extending toward the G9-G13 loop, it establishes ionic interactions between guanidinium group and the phosphate groups of T10 and G12. The other side chain displays an H-bond between the amide moiety and G18 and, by guanidinium group, establishes ionic interactions with phosphates of G16, G17, and G18.

**Figure 4:**
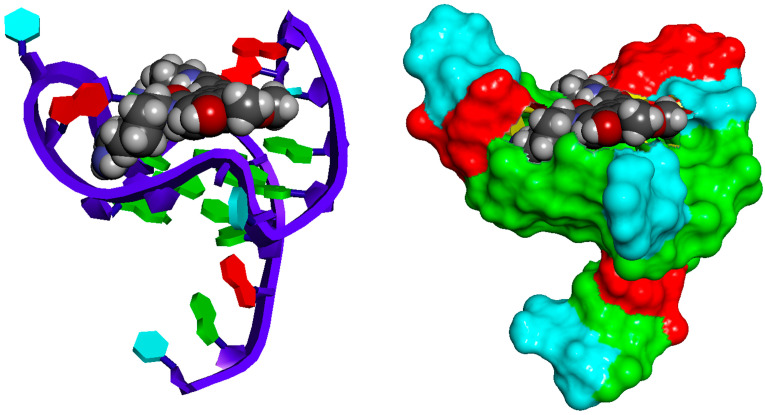
Lateral view (**left**) and docking site at 3′-end of **3** complexed with the Pu22T14T23. The nucleotides are shown as slabs and filled sugars: adenine in red, cytosine in yellow, guanine in green, and thymine in blue. The Pu22T14T23 is represented in CPK, the binding site is contoured by the solid solvent accessible surface (SAS), and the ligand is in CPK (**right**).

**Figure 5:**
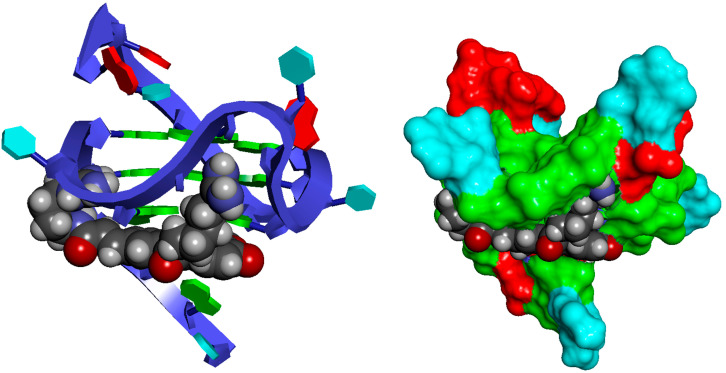
Lateral view (**left**) and docking site at 5′-end of **3** complexed with the Pu22T14T23 (**right**). The nucleotides are shown as slabs and filled sugars: adenine in red, cytosine in yellow, guanine in green, and thymine in blue. The Pu22T14T23 is represented in CPK, the binding site is contoured by the solid SAS (solvent accessible surface), and the ligand is in CPK.

**Figure 6:**
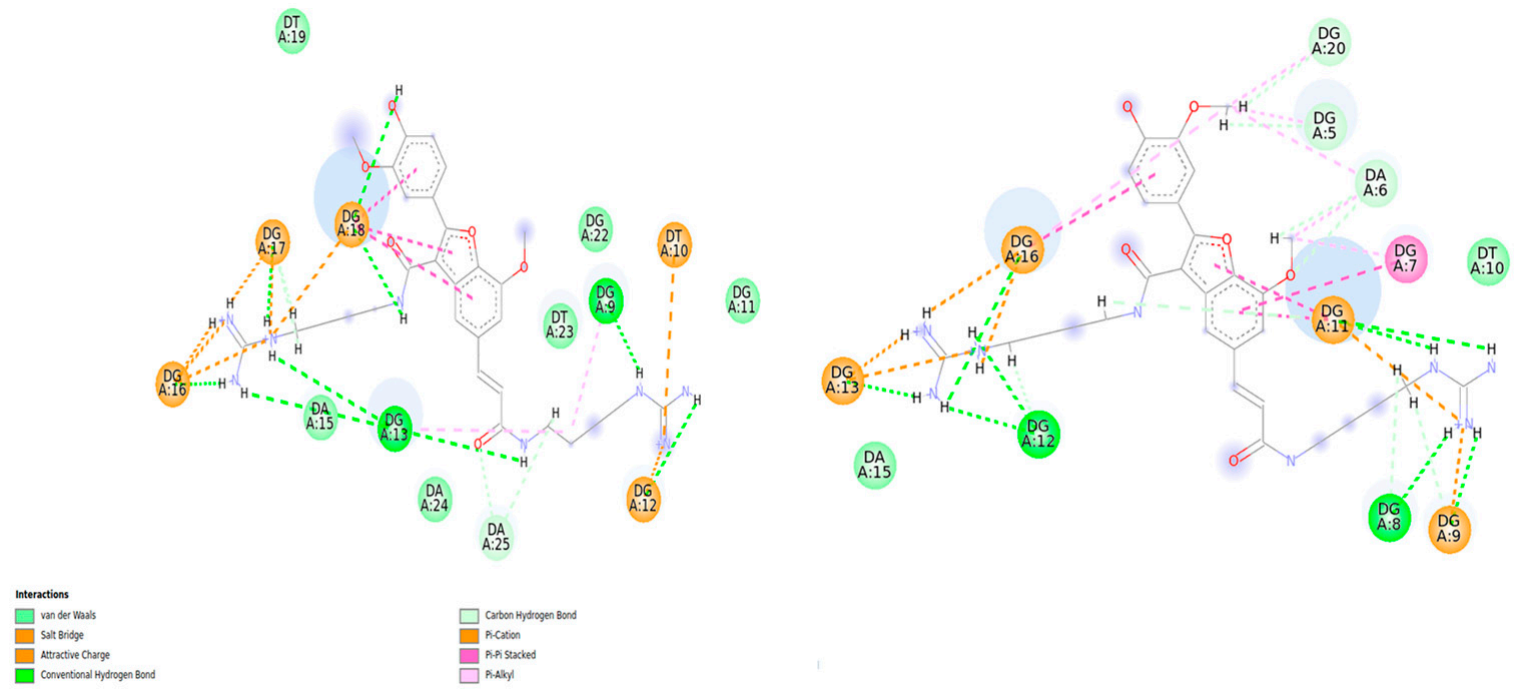
Two-dimensional representation of the molecular interactions between **3** and the Pu22T14T23. On the left 3′-end and on the right 5′-end binging site. Here, the colors used refer to the different interactions, as indicated in the legend.

At the 5′-end, the docking pose of **3** shows the planar benzofuran and phenol moieties stacking with the guanines G7, G11, and G16, respectively. Additionally, the side chains with the protonated guanidinium groups extend toward the groove defined by the G11-G16 tract, forming ionic interactions with the phosphate groups of G9, G11, G13, and G16 (Table 1). Moreover, the guanidinium establishes a web of H-bonds. The docking sites of **3** are shown in Figure 4 and Figure 5, while a detail of the environment of aromatic moiety and of its charged guanidinium group are shown in Figure 6. The solvation energy term contribution is −780.691 and −774.783 kcal/mol for 5′-end and 3′-end binding sites, respectively.

#### 2.2.2. Docking Analysis of the Interaction Between Pu22T14T23 and **4**

**Figure 7:**
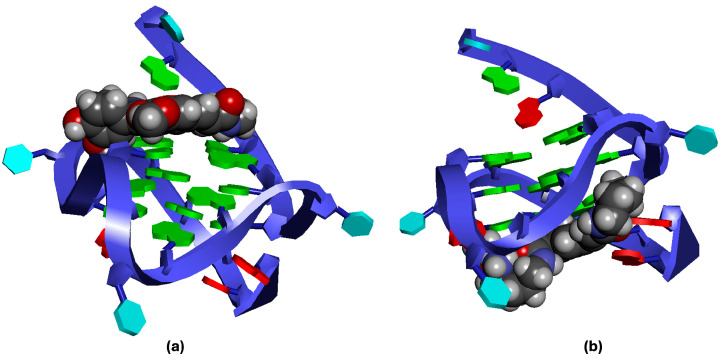
Side views of the **4** complex with the c-MYC G-quadruplex sequence at the 5′-end (**a**) and 3′-end (**b**). The aromatic moiety of the ligand is located near the center of the quadruplex, while the side chain fits into the groove between G17 and G20. The nucleotides are shown as follows: adenine in red, guanine in green, and thymine in blue.

**Figure 8:**
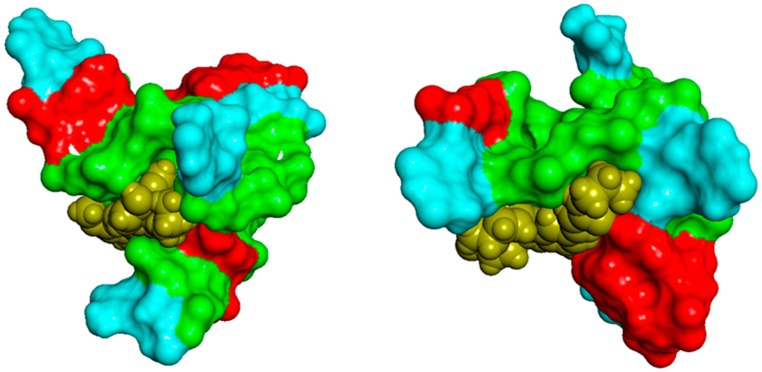
Docking sites of **4** on the left 5′-end and on the right 3′-end: the Pu22T14T23 is represented in CPK, the binding site is contoured by the solid SAS (solvent accessible surface), and the ligand is in CPK. The nucleotides are shown as follows: adenine in red, guanine in green, and thymine in blue.

**Figure 9:**
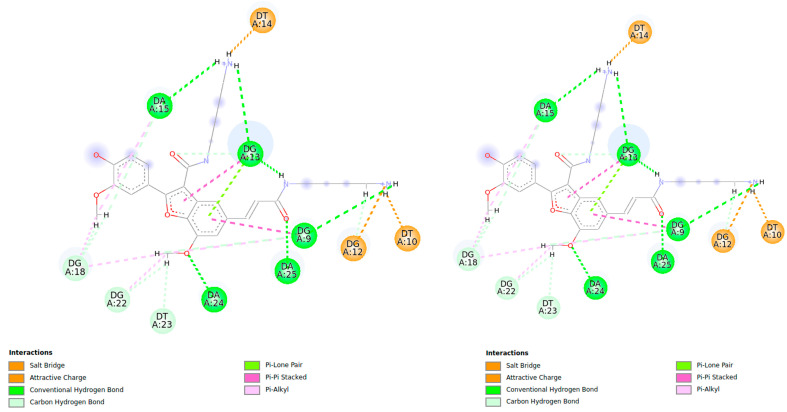
Two-dimensional representation of the molecular interactions between **4** and the Pu22T14T23, on the left 5′-end and on the right 3′-end binging site. Here, the colors used refer to the different interactions, as indicated in the legend.

In the case of **4**, the docking conformation was analyzed by 3D visualization (Figure 7 and Figure 8). Ligand **4** is capable of forming π–π stacking interactions with the guanines of G-quadruplex as well as it binds externally at the groove of quadruplex DNA. Specifically, docking experiments confirm the above cited NOEs interactions and show the involvement of G16 by π-π interactions as well as hydrogen bonds with G7 and G11 residues and π-cation with G20 and G17 residues at 5′-end. Moreover G9, G13, G18, and G22, belonging to 3′-end, show π-π and hydrogen bonds interactions. The methoxy group on the phenolic ring shows interactions with G16, whereas the methoxy group on the benzofuran ring shows interactions with G9, G12, G13, G18, G21, and G22. The 2D pattern diagram is shown in Figure 9. The solvation energy term contribution is −675.224 and −685.856 kcal/mol for 5′-end and 3′-end binding sites, respectively.

Overall, docking results were fully consistent with binding modes inferred by NMR data, confirming that **3** interacts better with G-quadruplex DNA compared to **4**. To obtain a more comprehensive view of the effect of the guanidinium group on the interaction with the G-quadruplex, we also performed docking studies on compound **1**, despite its weaker impact compared to compounds **3** and **4** on the displacement of NMR signals (see NMR experiments). In agreement with the NMR results, molecular docking studies indicate that compound **1** engages in electrostatic interactions with the phosphate oxygen atoms of the G-quadruplex, predominantly within the groove region. π–π stacking interactions with the guanine tetrads are also observed, although they appear to be less extensive compared to compounds **3** and **4** (Figures S6 and S7).

### 2.3. Cytotoxicity Evaluation

In vitro antiproliferative activity of compounds **1**–**4** was evaluated using the osteosarcoma U2OS cell line, which exhibits low levels of c-Myc expression [26], and the breast cancer MDA-MB-231 cell line, which, conversely, expresses c-Myc at high levels [27]. The acid **9** was tested to evaluate the role of the guanidinium moieties. The IC_50_ values were determined from dose–response curves as the concentration of compound required to inhibit cell growth by 50% (Table 2). RHPS4 was used as a reference compound. All experiments were performed in triplicate.

The cytotoxicity tests further confirmed the results obtained from the spectroscopic evaluation. Compound **3**, featuring a planar benzofuran ring connected to guanidinium-ending side chains, was the most active on U2OS cells and, even more, on MDA-MB-231 cells, the latter characterized by particularly high levels of c-Myc expression. Compound **1**—bearing the guanidinium moiety—still retained a similar antiproliferative activity in both cell lines. Conversely, the corresponding derivatives with a primary amino group in place of the guanidinium group (compounds **2** and **4**) did not show cytotoxic activity, as well as the acid **9**.

## 3. Materials and Methods

### 3.1. Chemistry

All reagents and solvents were purchased from commercial suppliers and used without further purification. Unless otherwise specified, chemicals were from Sigma-Aldrich (Milan, Italy) as was the horseradish peroxidase used for some of the synthetic procedures. All reactions requiring anhydrous conditions were performed under a positive nitrogen flow (after vacuuming the flask), and all glassware were oven-dried. The ^1^H NMR and ^13^C NMR spectra were recorded with an Avance NEO 400 (Bruker Italia S.R.L. Milan, Italy) (^1^H, 400 MHz, ^13^C, 101 MHz) spectrometer and a Varian Mercury 300 (Turin, Italy) (^1^H, 300 MHz) spectrometer, using the residual signal of the deuterated solvent as internal standard; (δ) are expressed in ppm (Supplementary Figures S8–S23). The coupling constants (*J*) are reported in Hz. For the NMR analysis, we used chloroform (CHCl_3_), acetone, dimethyl sulfoxide (DMSO), and methanol (MeOH) as deuterated solvents. All the spectra were recorded at room temperature (298.15K). ^1^H NMR signals are indicated with the following abbreviations: s (singlet), bs (broad singlet), d (doublet), t (triplet), dd (doublet of doublets), ddd (doublet of doublet of doublets), dt (double triplet), m (multiplet). TLC analyses were performed using commercial silica gel 60 F254 aluminum sheets. Reference spots were revealed under UV lamp (*λ* = 254 or 365 nm). Development reagents, such as ninhydrin (1.5 g of ninhydrin, 3.0 mL of acetic acid in 100 mL of ethanol), were used occasionally as needed. Isolation and purification of the products were performed by flash column chromatography on silica gel 60 (230–400 mesh) using Büchi Pump Manager (C-615 and C-601) (Büchi S.R.L. Cornaredo, Italy) equipment or by gravimetric chromatographic column.

#### 3.1.1. Synthetic Procedures

##### Synthesis of Ethyl (2*R*,3*R*)-5-((*E*)-3-Ethoxy-3-oxoprop-1-en-1-yl)-2-(4-hydroxy-3-methoxyphenyl)-7-methoxy-2,3-dihydrobenzofuran-3-carboxylate (**6**) and (2*R*,3*R*)-5-((*E*)-2-Carboxyvinyl)-2-(4-hydroxy-3-methoxyphenyl)-7-methoxy-2,3-dihydrobenzofuran-3-carboxylic acid (**7**)

Compounds **6** and **7** were obtained starting from ferulic acid **5**, converted into ethyl ester and undergone dimerization via HRP/H_2_O_2_, followed by basic hydrolysis [24,28].

##### Synthesis of (*E*)-5-(2-Carboxyvinyl)-2-(4-hydroxy-3-methoxyphenyl)-7-methoxybenzofuran-3-carboxylic acid (**9**)

To a solution of ethyl ferulate dimer **6** (255mg, 0.58 mmol, 1 eq), dichloromethane (3.2 mL), triethylamine (175 mg, 1.74 mmol, 3 eq), and acetic anhydride (88 mg, 0.87 mmol, 1.5 eq) were added. The reaction was stirred at room temperature overnight, then diluted with dichloromethane and washed with water, 1 N HCl, and brine. The organic phase was dried over anhydrous Na_2_SO_4_, filtered, and concentrated under reduced pressure to give ethyl (*E*)-2-(4-acetoxy-3-methoxyphenyl)-5-(3-ethoxy-3-oxoprop-1-en-1-yl)-7-methoxy-2,3-dihydrobenzofuran-3-carboxylate as a white solid in 88% yield. ^1^H NMR (400 MHz, acetone-*d_6_*) δ 7.63 (d, *J* = 15.9 Hz, 1H), 7.35 (d, *J* = 1.6 Hz, 1H), 7.30 (d, *J* = 1.4 Hz, 1H), 7.24 (d, *J* = 1.8 Hz, 1H), 7.08 (d, *J* = 8.1 Hz, 1H), 7.03 (dd, *J* = 8.1 Hz, 1.8 Hz, 1H), 6.43 (d, *J* = 15.9 Hz, 1H), 6.14 (d, *J* = 7.7 Hz, 1H), 4.47 (d, *J* = 7.7, H), 4.36–4.23 (m, 2H), 4.20 (q, *J* = 7.1 Hz, 2H), 3.94 (s, 3H), 3.83 (s, 3H), 2.24 (s, 3H), 1.30 (m, 6H). ^13^C NMR (100 MHz, acetone-*d_6_*) δ 170.98, 168.95, 167.27, 152.56, 150.84, 145.89, 145.18, 141.09, 139.76, 129.76, 127.13, 123.92, 119.03, 116.88, 113.43, 111.35, 87.60, 62.35, 60.60, 56.55, 56.32, 56.16, 20.48, 14.66, 14.50.

To a solution of the abovementioned intermediate (1.0 g, 2.1 mmol, 1 eq) in toluene (147 mL), DDQ (9.37 mg, 41.3 mmol, 20 eq) was added. The reaction was refluxed at 100 °C for 30 h, then cooled at room temperature, filtered on celite, and evaporated under reduced pressure. The mixture was diluted with ethyl acetate, washed with water and a solution 5% of NaHCO_3_. The organic phase was dried over anhydrous Na_2_SO_4_, filtered and concentrated under reduced pressure. Purification by column chromatography (cyclohexane/ethyl acetate 8:2 to 5:5) gave the desired product **8** as a purple solid in 78% yield. ^1^H NMR (400 MHz, methanol-*d_4_*) δ 9.19 (d, *J* = 2.0 Hz, 1H), 9.15 (d, *J* = 1.4 Hz, 1H), 9.05 (d, *J* = 16.0 Hz, 1H), 8.93 (dd, *J* = 8.3, 2.0 Hz, 1H), 8.70 (d, *J* = 1.4 Hz, 1H), 8.51 (d, *J* = 8.3 Hz, 1H), 7.86 (d, *J* = 16.0 Hz, 1H), 5.70 (q, *J* = 7.1 Hz, 2H), 5.50 (q, *J* = 7.1 Hz, 2H), 5.38 (s, 3H), 5.20 (s, 3H), 3.56 (s, 3H), 2.67 (t, *J* = 7.1 Hz, 3H), 2.57 (t, *J* = 7.1 Hz, 3H). ^13^C NMR (100 MHz, methanol-*d_4_*) δ 168.82, 167.11, 163.93, 161.01, 151.97, 146.53, 145.66, 142.78, 132.86, 129.90, 128.60, 123.63, 123.01, 118.75, 117.00, 114.82, 107.12, 61.58, 60.78, 56.67, 56.53, 20.52, 14.66, 14.55.

Compound **8** (770 mg, 1.6 mmol, 1 eq) was dissolved in dioxane (38 mL) and cooled at 0 °C. A 2 M solution of NaOH (77 mL, 96 eq) was added, and the reaction was allowed to reach room temperature. After 20 h, the reaction was acidified with concentrated HCl (*w/v*, pH = 1) and extracted with ethyl acetate. The organic phase was dried over anhydrous Na_2_SO_4_, filtered and evaporated under reduced pressure to give the desired product **9** as a brownish solid in quantitative yield. ^1^H NMR (400 MHz, DMSO-*d_6_*) δ 7.76 (d, *J* = 1.3 Hz, 1H), 7.72–7.67 (m, 2H), 7.50 (dd, *J* = 8.4, 2.1 Hz, 1H), 7.40 (d, *J* = 1.3 Hz, 1H), 6.93 (d, *J* = 8.4 Hz, 1H), 6.59 (d, *J* = 15.9 Hz, 1H), 4.03 (s, 3H), 3.84 (s, 3H). ^13^C NMR (100 MHz, DMSO-*d_6_*) δ 167.64, 164.65, 160.69, 149.18, 147.03, 144.94, 144.57, 143.07, 131.43, 129.22, 122.90, 119.64, 118.70, 115.54, 115.27, 113.41, 107.97, 106.22, 56.16, 55.69.

##### General Procedure for the Synthesis of Compounds (2*R*,3*R*)-*N*-(4-Guanidinobutyl)-5-((*E*)-3-((4-guanidinobutyl)amino)-3-oxoprop-1-en-1-yl)-2-(4-hydroxy-3-methoxyphenyl)-7-methoxy-2,3-dihydrobenzofuran-3-carboxamide (**1**) and (*E*)-*N*-(4-Guanidinobutyl)-5-(3-((4-guanidinobutyl)amino)-3-oxoprop-1-en-1-yl)-2-(4-hydroxy-3-methoxyphenyl)-7-methoxybenzofuran-3-carboxamide (**3**)

Agmatine functionalized dimer 1 was obtained following already reported protocols starting from dihydrobenzofuran dimer **7** [29,30]. The same procedure was applied to benzofuran dimer 9.

To a solution of the selected dimer (**7** or **9**, 1 eq) and **10a** (3 eq) in dry DMF (0.05 M) under nitrogen atmosphere, EDC HCl (3 eq) and HOBt (3 eq) were added. The reaction was stirred at room temperature for 24 h, then diluted with EtOAc and washed with 1 N HCl, 5% aqueous NaHCO_3_, and brine. The organic phase was dried over anhydrous Na_2_SO_4_, filtered and evaporated under reduced pressure.

##### Tetra-Boc-Protected (2*R*,3*R*)-*N*-(4-Guanidinobutyl)-5-((*E*)-3-((4-guanidinobutyl)amino)-3-oxoprop-1-en-1-yl)-2-(4-hydroxy-3-methoxyphenyl)-7-methoxy-2,3-dihydrobenzofuran-3-carboxamide) (**11a**)

Purification by column chromatography (cyclohexane/ethyl acetate 3:7 to 1:9) gave **11a** as a white solid in 46% yield. ^1^H NMR (400 MHz, acetone-*d_6_*) δ 11.64 (s, 2H), 8.32 (s, 2H), 7.96 (s, 3H), 7.65–7.59 (m, 1H), 7.45 (d, *J* = 15.6 Hz, 1H), 7.35–7.26 (m, 1H), 7.12 (s, 1H), 7.10 (s, 1H), 7.01 (d, *J* = 1.8 Hz, 1H), 6.86 (dd, *J* = 8.2, 1.8 Hz, 1H), 6.83 (d, *J* = 8.1 Hz, 1H), 6.51 (d, *J* = 15.6 Hz, 1H), 6.02 (d, *J* = 8.2 Hz, 1H), 4.26 (d, *J* = 8.2 Hz, 1H), 3.88 (s, 3H), 3.83 (s, 3H), 3.49–3.25 (m, 8H), 1.68–1.57 (m, 8H), 1.51 (s, 18H), 1.43 (s, 9H), 1.42 (s, 9H). ^13^C NMR (100 MHz, acetone-*d_6_*) δ 170.62, 166.19, 164.62, 162.73, 156.95, 153.83, 150.54, 148.52, 147.74, 145.56, 140.12, 132.75, 130.15, 129.59, 120.64, 119.83, 117.26, 115.88, 112.99, 110.57, 89.24, 83.67, 83.64, 78.89, 78.86, 58.24, 56.42, 56.34, 41.04, 40.95, 39.93, 39.58, 28.51, 28.15, 27.64, 27.53, 27.33.

##### Tetra-Boc-Protected (*E*)-*N*-(4-Guanidinobutyl)-5-(3-((4-guanidinobutyl)amino)-3-oxoprop-1-en-1-yl)-2-(4-hydroxy-3-methoxyphenyl)-7-methoxybenzofuran-3-carboxamide (**12a**)

Purification by column chromatography (ethyl acetate 100%) gave **12a** as a white solid in 35% yield. ^1^H NMR (400 MHz, acetone-*d_6_*) δ 11.73–11.59 (m, 2H), 8.42–8.28 (m, 2H), 8.26–8.07 (m, 1H), 7.64–7.57 (m, 2H), 7.55 (d, *J* = 1.0 Hz, 1H), 7.53 (t, 1H), 7.47 (dd, *J* = 8.3, 2.0 Hz, 1H), 7.45 (t, 1H), 7.15 (d, *J* = 1.1 Hz, 1H), 6.99 (d, *J* = 8.3 Hz, 1H), 6.69 (d, *J* = 15.6 Hz, 1H), 4.07 (s, 3H), 3.94 (s, 3H), 3.60–3.35 (m, 8H), 1.76–1.62 (m, 8H), 1.52 (s, 18H), 1.49–1.36 (m, 18H). ^13^C NMR (100 MHz, acetone-*d_6_*) δ 166.02, 164.66, 164.62, 164.21, 156.97, 156.93, 155.97, 153.84, 149.36, 148.41, 146.16, 144.03, 140.43, 133.01, 131.11, 122.45, 122.34, 121.97, 116.21, 113.48, 113.38, 112.06, 107.36, 83.64, 78.88, 78.82, 56.50, 56.46, 41.05, 40.95, 39.79, 39.63, 28.52, 28.46, 28.16, 27.65, 27.62, 27.56, 27.44.

A solution of 20% TFA (40 eq) in DCM was added dropwise to Boc-protected intermediate (**11a** or **12a**, 1 eq) at 0 °C. The reaction was stirred at room temperature for 3 h, then evaporated under reduced pressure.

##### (2*R*,3*R*)-*N*-(4-Guanidinobutyl)-5-((*E*)-3-((4-guanidinobutyl)amino)-3-oxoprop-1-en-1-yl)-2-(4-hydroxy-3-methoxyphenyl)-7-methoxy-2,3-dihydrobenzofuran-3-carboxamide (rac-Hordatine C, **1**)

Precipitation from diethyl ether gave the desired product **1** as a white solid in quantitative yield. [M+2H]**^2+^** 306.1678. ^1^H NMR (400 MHz, methanol-*d_4_*) δ 7.47 (d, *J* = 15.7 Hz, 1H), 7.18 (d, *J* = 1.6 Hz, 1H), 7.00 (s, 1H), 6.95 (s, 1H), 6.81 (s, 2H), 6.49 (d, *J* = 15.7 Hz, 1H), 5.95 (d, *J* = 8.1 Hz, 1H), 4.24 (d, *J* = 8.2 Hz, 1H), 3.92 (s, 3H), 3.84 (s, 3H), 3.38–3.29 (m, 4H), 3.27–3.14 (m, 4H), 1.63 (m, 8H). ^13^C NMR (101 MHz, methanol-*d_4_*) δ 173.22, 169.15, 158.61, 151.36, 149.33, 148.26, 146.22, 141.75, 132.56, 130.47, 129.48, 120.04, 119.49, 118.28, 116.36, 112.86, 110.57, 89.97, 58.72, 56.83, 56.44, 42.09, 42.01, 40.02, 39.84, 28.13, 27.78, 27.20.

##### (*E*)-*N*-(4-Guanidinobutyl)-5-(3-((4-guanidinobutyl)amino)-3-oxoprop-1-en-1-yl)-2-(4-hydroxy-3-methoxyphenyl)-7-methoxybenzofuran-3-carboxamide (**3**)

Precipitation from diethyl ether gave the desired product **3** as a white solid in quantitative yield. [M+2H]^2+^ 305.1601. ^1^H NMR (400 MHz, methanol-*d_4_*) δ 7.58 (d, J = 15.7 Hz, 1H), 7.47 (d, J = 2.0 Hz, 1H), 7.36 (dd, J = 8.3, 2.1 Hz, 1H), 7.33 (d, J = 1.5 Hz, 1H), 7.12 (d, J = 1.5 Hz, 1H), 6.90 (d, J = 8.3 Hz, 1H), 6.61 (d, J = 15.8 Hz, 1H), 4.05 (s, 3H), 3.92 (s, 3H), 3.49–3.42 (m, 2H), 3.35 (m, 2H), 3.25 (m, 4H), 1.68 (m, 8H). ^13^C NMR (101 MHz, methanol-*d_4_*) δ 168.90, 166.99, 158.68, 156.66, 149.90, 149.16, 146.81, 144.86, 142.16, 133.08, 131.09, 122.34, 121.92, 121.13, 116.57, 114.25, 112.75, 111.98, 106.59, 56.70, 56.59, 42.10, 40.24, 39.88, 27.73, 27.63, 27.49, 27.21.

##### General Procedure for the Synthesis of (2*R*,3*R*)-*N*-(4-Aminobutyl)-5-((*E*)-3-((4-aminobutyl)amino)-3-oxoprop-1-en-1-yl)-2-(4-hydroxy-3-methoxyphenyl)-7-methoxy-2,3-dihydrobenzofuran-3-carboxamide (**2**) and (*E*)-*N*-(4-Aminobutyl)-5-(3-((4-aminobutyl)amino)-3-oxoprop-1-en-1-yl)-2-(4-hydroxy-3-methoxyphenyl)-7-methoxybenzofuran-3-carboxamide (**4**)

To a solution of the dimer (7 or 9, 1 eq) and 10b (3 eq) in dry DMF (0.05 M) under nitrogen atmosphere, EDC HCl (3 eq) and HOBt (3 eq) were added. The reaction was stirred at room temperature for 24 h, then diluted with EtOAc and washed with 1 N HCl, 5% aqueous NaHCO_3_ and brine. The organic phase was dried over anhydrous Na_2_SO_4_, filtered and evaporated under reduced pressure.

##### Bis-Boc-Protected (2*R*,3*R*)-*N*-(4-Aminobutyl)-5-((*E*)-3-((4-aminobutyl)amino)-3-oxoprop-1-en-1-yl)-2-(4-hydroxy-3-methoxyphenyl)-7-methoxy-2,3-dihydrobenzofuran-3-carboxamide (**11b**)

Purification by column chromatography (ethyl acetate 100%) gave the desired product **11b** as a white solid in 54% yield. ^1^H NMR (400 MHz, acetone-*d_6_*) δ 7.96 (s, 1H), 7.73 (s, 1H), 7.63 (t, *J* = 5.7 Hz, 1H), 7.45 (d, *J* = 15.6 Hz, 1H), 7.29 (t, *J* = 6.1 Hz, 1H), 7.10 (s, 2H), 7.01 (d, *J* = 1.7 Hz, 1H), 6.86 (dd, *J* = 8.2, 1.7 Hz, 1H), 6.83 (d, *J* = 8.1 Hz, 1H), 6.50 (d, *J* = 15.6 Hz, 1H), 6.03 (d, *J* = 8.3 Hz, 1H), 4.25 (d, *J* = 8.3 Hz, 1H), 3.88 (s, 3H), 3.83 (s, 3H), 3.41–3.21 (m, 4H), 3.09 (q, *J* = 6.3 Hz, 4H), 1.65–1.48 (m, 8H), 1.39 (s, 18H). ^13^C NMR (100 MHz, acetone-*d_6_*) δ 170.56, 166.26, 156.88, 156.72 150.46, 148.52, 147.74, 145.54, 140.15, 132.75, 130.11, 129.66, 120.55, 119.82, 116.92, 115.91, 113.17, 110.58, 89.13, 78.52, 78.36, 58.14, 56.41, 56.34, 40.82, 40.03, 39.66, 28.68, 28.35, 27.82, 27.74.

##### Bis-Boc-Protected (*E*)-*N*-(4-Aminobutyl)-5-(3-((4-aminobutyl)amino)-3-oxoprop-1-en-1-yl)-2-(4-hydroxy-3-methoxyphenyl)-7-methoxybenzofuran-3-carboxamide (**12b**)

Purification by column chromatography (ethyl acetate 100%) gave the desired product **12b** as a white solid in 33% yield. ^1^H NMR (400 MHz, acetone-*d_6_*) δ 8.26–8.13 (m, 1H), 7.65 (d, *J* = 1.9 Hz, 1H), 7.59 (d, *J* = 15.6 Hz, 1H), 7.56–7.51 (m, 2H), 7.49 (dd, *J* = 8.3, 2.0 Hz, 1H), 7.43–7.33 (m, 1H), 7.14 (d, *J* = 1.2 Hz, 1H), 6.99 (d, *J* = 8.3 Hz, 1H), 6.68 (d, *J* = 15.6 Hz, 1H), 6.09–5.95 (m, 2H), 4.07 (s, 3H), 3.94 (s, 3H), 3.50 (q, *J* = 6.6 Hz, 2H), 3.42–3.29 (m, 2H), 3.21–3.06 (m, 4H), 1.77–1.51 (m, 8H), 1.41 (s, 18H). ^13^C NMR (100 MHz, acetone-*d_6_*) δ 166.05, 164.25, 156.75, 155.85, 149.34, 148.41, 146.14, 143.97, 140.40, 132.99, 131.11, 122.41, 122.21, 122.01, 116.20, 113.46, 113.13, 112.01, 107.53, 107.46, 78.47, 78.37, 56.49, 56.45, 40.82, 40.04, 39.69, 28.68, 28.50, 28.36, 27.78.

A solution of 20% TFA (20 eq) in DCM was added dropwise to Boc-protected intermediate (**11b** or **12b**, 1 eq) at 0 °C. The reaction was stirred at room temperature for 3 h, then evaporated under reduced pressure.

##### (2*R*,3*R*)-N-(4-Aminobutyl)-5-((*E*)-3-((4-aminobutyl)amino)-3-oxoprop-1-en-1-yl)-2-(4-hydroxy-3-methoxyphenyl)-7-methoxy-2,3-dihydrobenzofuran-3-carboxamide (**2**)

Precipitation from diethyl ether gave the desired product 2 as a white solid in quantitative yield. [M+2H]^2+^ 264.1462. ^1^H NMR (400 MHz, methanol-*d_4_*) δ 7.47 (d, *J* = 15.7 Hz, 1H), 7.17 (d, *J* = 1.6 Hz, 1H), 7.01 (d, *J* = 1.4 Hz, 1H), 6.94 (d, *J* = 1.2 Hz, 1H), 6.81 (d, *J* = 1.1 Hz, 2H), 6.48 (d, *J* = 15.6 Hz, 1H), 5.95 (d, *J* = 8.0 Hz, 1H), 4.23 (d, *J* = 8.0 Hz, 1H), 3.92 (s, 3H), 3.83 (s, 3H), 3.38–3.31 (m, 4H), 2.99–2.93 (m, 4H), 1.77–1.59 (m, 8H). ^13^C NMR (100 MHz, methanol-*d_4_*) δ 173.26, 169.13, 151.38, 149.05, 146.22, 141.79, 132.60, 130.47, 129.41, 120.07, 119.47, 118.26, 116.37, 112.94, 110.60, 89.94, 58.65, 56.83, 56.45, 40.35, 40.26, 39.99, 39.66, 27.53, 25.90.

##### (*E*)-*N*-(4-Aminobutyl)-5-(3-((4-aminobutyl)amino)-3-oxoprop-1-en-1-yl)-2-(4-hydroxy-3-methoxyphenyl)-7-methoxybenzofuran-3-carboxamide (**4**)

Purification from diethyl ether gave the desired product **4** as a white solid in quantitative yield. [M+2H]^2+^ 263.1384. ^1^H NMR (400 MHz, methanol-*d_4_*) δ 7.62 (d, *J* = 15.7 Hz, 1H), 7.49 (d, *J* = 2.0 Hz, 1H), 7.41–7.36 (m, 2H), 7.16 (d, *J* = 1.4 Hz, 1H), 6.92 (d, *J* = 8.3 Hz, 1H), 6.63 (d, *J* = 15.7 Hz, 1H), 4.07 (s, 3H), 3.94 (s, 3H), 3.47 (t, *J* = 6.2 Hz, 2H), 3.38 (t, *J* = 6.5 Hz, 2H), 3.00 (q, *J* = 6.7 Hz, 4H), 1.85–1.56 (m, 8H). ^13^C NMR (100 MHz, methanol-*d_4_*) δ 168.90, 167.03, 156.84, 150.01, 149.20, 146.87, 144.93, 142.25, 133.13, 131.12, 122.43, 121.89, 121.13, 116.60, 114.30, 112.70, 112.05, 106.60, 56.72, 56.61, 40.35, 40.12, 39.70, 27.53, 27.41, 26.13, 25.94.

### 3.2. Cytotoxicity Assay

Cytotoxic activity of the compounds was evaluated using the human osteosarcoma U2OS (ATCC HTB-96) and breast cancer MDA-MB-231 (HTB-26) cell line. Cells were cultured in McCoy’s 5A (U2OS cells) or RPMI1640 (MDA-MB-231 cells) medium supplemented with 10% fetal bovine serum at 37 °C in a 5% CO_2_ atmosphere. Cells were seeded into 24-well plates (35,000 cells/well for U2OS and 50,000 cells/well for MDA-MB-231 cells) and, after 24 h, treated with the compounds at concentrations ranging from 1 to 100 µM. After 72 h of treatment, cells were harvested and counted using a cell counter (ZB1, Beckman Coulter, CA, USA). The IC_50_ values were determined from dose–response curves as the concentration of compound required to inhibit cell growth by 50%. RHPS4 was used as a reference compound. All experiments were performed in triplicate.

### 3.3. NMR Studies

A Bruker Avance 600 MHz spectrometer (Milan, Italy) available at the Unitech Cospect platform (University of Milan) was used to measure NMR spectra. A 5 mm triple-resonance probe with z-axis gradients and a variable temperature control unit was used. G-quadruplex Pu22T14T23–70 OD, 0.55 mL H_2_O/D_2_O 9:1 buffer solution having 70 mM KCl and 25 mM potassium phosphate, pH = 7.0, 25 °C) was characterized, following the previous assignments [13,31]. Water suppression was achieved by excitation sculpting sequence from standard Bruker library. The oligonucleotide was heated to 85 °C for 1 min and then cooled at room temperature overnight. For interaction studies with compounds **1**–**4**, a stock solution of drug was prepared in DMSO-*d_6_* at a concentration ranging from 35 mM to 50 mM. ^1^H NMR titrations were performed by adding increasing amounts of the drug to the oligonucleotide solution until R = [ligand]/[Pu22] = 2.0 was reached. The concentration of the DMSO in the sample solution was below 10% to avoid affecting the G4 conformation. Phase sensitive NOESY spectra were acquired at 25 °C in TPPI mode, with 2048 × 1024 complex FIDs, t_mix_ = 250 ms. TOCSY spectra were acquire with 80 ms of mixing time. The spectra were transformed and weighed with a 90° shifted sine-bell squared function to 4 K × 4 K real data points.

### 3.4. Docking Studies

The raw structural model of Pu22 was obtained from the RCSB Protein Data Bank (PDB access code: 2L7V) [25], the molecular structure of compounds **1**, **2**, and **3** was created using Avogadro 1.101.0 software [32] while the Structure version of the YASARA 24.4.10 (Yet Another Scientific Artificial Reality Application) software [33] was used to parameterize the starting molecular systems and build the ligand-Pu22 complexes, via molecular docking and subsequent optimization of the geometry of the resulting best poses. In particular, the interactions of 1, 3, and 4 with the Pu22 model were analyzed using the AutoDock Vina 1.25 [34] molecular docking software, as implemented in the YASARA Structure software. The molecular docking experiments were performed using the dock_run.mcr macro from YASARA Structure, with 100 docking runs, setting the number of binding modes to 10 and the exhaustiveness of search to 8, with a maximum energy difference of 3 kcal/mol. Each docking run was ranked based on the best Autodock Vina score values (kcal/mol) and the resulting poses were analyzed and the best one was chosen based on both the molecular docking score values and the compliance with experimental (NMR) data. For each ligand, the best pose was then optimized at the molecular mechanics level of theory (MM) by using the steepest descent conjugate method as implemented in the YASARA Structure software. In all of the experiments conducted (molecular docking and molecular geometry optimization), the systems were parameterized using the OL15 modification of the original Amber force field [35] as implemented in YASARA Structure. The resulting YOB files were converted into PDB format and used to analyze the position of each ligand and contact bases through three-dimensional visualization with BIOVIA Discovery Studio (DS) 2021visualizer [36].

## 4. Conclusions

The guanidine unit can be found in a wide range of natural products as well as pharmacologically active molecules. Guanidine-bearing compounds have been attracting considerable interest among researchers throughout the years whereby the guanidine moiety is one of the most widely used scaffolds in the design and synthesis of new bioactive compounds. In the search of novel chemotypes targeting the G4 structure within the *c-MYC* promoter, we focused on plant derived natural products containing a dihydrobenzofuran core—namely, the hordatines. Our interest in these compounds was sparked by the presence of the guanidinium group in their structure, which could potentially enhance G4 stabilization through electrostatic interactions with the phosphate backbone of DNA.

We initially evaluated the ability of hordatine **1** to stabilize the G-quadruplex structure located in the promoter region of the *c-MYC* oncogene by NMR spectroscopy. The results evidenced (highlighted) that the guanidinium group effectively contributes to G-quadruplex stabilization. In fact, derivative **2** that retains the molecular scaffold of compound **1**, with identical carbon chain lengths, but replaces the guanidinium moiety with an amino group shows a lower (weaker) interaction. The evaluation of analogues of compounds **1** and **2** in which the dihydrobenzofuran core was replaced by a benzofuran (compounds **3** and **4**, respectively) confirmed that a planar aromatic system enhances π–π stacking with the G-quartets, thereby improving binding affinity and stabilization.

The findings suggest that compounds **3** binds to the Pu22 quadruplex, stacking at the 3′-end with G18 and forming hydrogen and ionic bonds with nearby residues and phosphates, with one side chain interacting with G13 and G12, and the other with G18, G16, and G17. At the 5′-end, the benzofuran and phenol groups stack with G7, G11, and G16, while the side chains with guanidinium groups interact with the groove, forming ionic and hydrogen bonds with nearby phosphates and residues. Ligand **4** can form π–π stacking interactions with guanines in the G-quadruplex and bind externally at the groove. Docking experiments confirmed these interactions, involving G16 through π–π and hydrogen bonds with G7 and G11, and π-cation interactions with G20 and G17 at the 5′-end. Additionally, G9, G13, G18, and G22 at the 3′-end show π–π and hydrogen bond interactions. The methoxy group on the phenolic ring interacts with G16, while the methoxy on the benzofuran ring interacts with G9, G12, G13, G18, G21, and G22. Docking calculations supported the NMR results and allowed us to propose the structures of *c-Myc*/ligands complexes for compound **1**, **3**, and **4**, confirming the role of the guanidinium group with the DNA.

The in vitro antiproliferative activity of compounds **1**–**4** in osteosarcoma U2OS and breast cancer MDA-MB-231 cell lines further confirmed the results obtained by spectroscopic evaluation. Compound **3** with a planar benzofuran ring connected to guanidinium-ending side chains had the most promising IC_50_ in the micromolar range on both cell lines, endowed with high levels of c-Myc [26,27]. The design of novel guanidinium-substituted dimeric and monomeric derivatives of ferulic acid has been now actively undertaken in our laboratories with the aim of synthesizing more active and selective DNA-binding ligands. The molecular models here built for the selected compounds with G-quadruplex structures are indeed a precious source of inspiration for an effective optimization process, taking into account the unique features of the ligands in their bound conformations.

## Figures and Tables

**Table 1:** Intermolecular NOE interactions between **3** and Pu22 in the complex.

Compound 3	Pu22T14T23 3’-End	d(Å) from Docking Studies
Har (*o* OCH_3_ bf) ^a^	NHG18	4.28
Har (*o* OCH_3_ bf) ^a^	NHG22	4.13
OCH_3_ bf	NHG22	4.77
Har (*o* OCH_3_ on Phe-OH)	H8G22	5.39
Har (*p* OCH_3_ bf)	NHG13	3.10
	5’-end	
OCH_3_ bf	NHG7	3.65
OCH_3_	NHG20	4.94

^a^ bf = benzofuran moiety.

**Table 2:** Evaluation of the cytotoxicity of compounds **1**–**4**, as well as acid **9**, on osteosarcoma U2OS and breast cancer MDA-MB-231 cell lines.

	IC_50_ (µM, 72 h)	
Compound	U2OS	MDA-MD-231
RHPS4	4.7 ± 0.84	11.2 ± 7.4
**1**	93 ± 9	89 ± 5.2
**2**	>100	>100
**3**	56.9 ± 0.07	44 ± 5.6
**4**	>100	>100
**9**	>100	>100

## Data Availability

The original contributions presented in this study are included in the article/Supplementary Materials. Further inquiries can be directed to the corresponding author.

## Supplementary Materials

The following supporting information can be downloaded at: https://www.mdpi.com/article/10.3390/ijms262110580/s1.
