# Supplementary material for: Exploring Guanidinium Group Involvement in Hordatine Interactions with the G-Quadruplex Motif Within the c-MYC Promoter Region
Source: Int J Mol Sci. 2025 Oct 30;26(21):10580. doi: 10.3390/ijms262110580 (PMC12607513; doi:10.3390/ijms262110580)
Supplement: Supplementary file 1 [file ijms-26-10580-s001.zip › ijms-3886808-supplementary.pdf]

Exploring Guanidinium Group Involvement in Hordatine Interactions with the G-Quadruplex Motif Within the c-MYC Promoter Region

Denise Dozio [1], Aziza Caccia [1], Sabrina Dallavalle [1], Giovanni Luca Beretta [2], Paola Perego [2], Roberto Artali [3], Stefania Mazzini [1,*] and Salvatore Princiotto [1]

[1] Department of Food, Environmental and Nutritional Sciences (DeFENS), Università degli Studi di Milano, Via Celoria 2, 20133 Milan, Italy; denise.dozio@unimi.it (D.D.); aziza.caccia@unimi.it (A.C.); sabrina.dallavalle@unimi.it (S.D.); salvatore.princiotto@unimi.it (S.P.)
[2] Molecular Pharmacology Unit, Department of Experimental Oncology, Fondazione IRCCS Istituto Nazionale Tumori, Via Amadeo 42, 20133 Milan, Italy; giovanni.beretta@istitutotumori.mi.it (G.L.B.); paola.perego@istitutotumori.mi.it (P.P.)
[3] Scientia Advice di Roberto Artali, 20832 Desio, Italy; roberto.artali@scientia-advice.com
* Correspondence: stefania.mazzini@unimi.it

Table S1. Selected chemical shift values of the complex **4**/Pu22T14T23 Pag. S2

Table S2. Selected chemical shift values of the complex **3**/Pu22T14T23 Pag. S3

Table S3. Selected chemical shift values of the complex **1**/ Pu22T14T23 Pag. S4

Figure S1. Imino proton region of the 1D NMR titration spectra of Pu22 with **2** Pag. S5

Figure S2. Imino proton region of the 1D NMR titration spectra of Pu22 with **1** Pag. S5

Figure S3. Aromatic protons region of the 1D NMR titration spectra of Pu22 with **4** and **3** Pag. S6

Figure S4. Selected region of the 2D-NOESY spectrum (compound **4**) Pag. S6

Figure S5. Selected region of the 2D-NOESY spectrum (compound **3**) Pag. S7

Figure S6. Molecular interactions between **1** and the Pu22T14T23 Pag. S7

Figure S7. Complex of **1** with the c-MYC G-quadruplex sequence Pag. S8

Figure S8–S23. ^{1}H NMR and ^{13}C NMR spectra Pag. S9

Table S1. Selected chemical shift values of the complex **4**/Pu22T14T23.

	**H2/NH/Me**	***Δδ*[a]**	**H2/NH/Me**	***Δδ*[b]**
T4	1.51	-0.09	1.51	-0.09
G7	11.56	-0.11	11.53	**- 0.14**
G8	11.08	-0.07	11.08	- 0.07
G9	10.66	+0.13	10.69	**+ 0.16**
T10	1.92	0.00	1.92	0.00
G11	11.36	-0.27	11.20	**- 0.43**
G12	11.26	-0.15	11.21	- 0.20
G13	10.93	-0.05	10.91	- 0.07
T14	1.87	-0.02	1.87	-0.02
A15	-	-	8.22	-0.07
G16	11.61	-0.21	11.53	**- 0.29**
G17	11.08	-0.09	11.08	- 0.09
G18	10.83	-0.13	10.78	**- 0.18**
T19	1.92	0.00	1.92	0.00
G20	11.17	-0.03	11.15	- 0.05
G21	11.22	-0.06	11.20	- 0.08
G22	10.88	-0.10	10.77	**- 0.21**
T23	1.66	+0.25	1.66	+0.25

[a] $\Delta\delta = \delta_{bound}-\delta_{free.}$ R = 1.0; [b] $\Delta\delta = \delta_{bound}-\delta_{free.}$ R = 2.0.

Table S2. Selected chemical shift values of the complex **3**/ Pu22T14T23.

	H2/NH/Me	*Δδ*[a]	H2/NH/Me	*Δδ*[b]	H6/H8	H1′
T4	1.51	-0.09	1.51	-0.09	7.15	5.82
G7	11.55	-0.12	11.52	**- 0.15**	7.88	5.95
G8	11.08	-0.07	11.05	- 0.10	7.63	6.04
G9	10.61	+0.08	10.64	**+ 0.11**	7.65	
T10	1.92	0.00	1.92	0.00	7.85	6.42
G11	11.42	-0.21	11.31	**- 0.32**	7.85	5.74
G12	11.29	-0.12	11.22	- 0.19	7.35	5.84
G13	10.92	-0.06	10.88	- 0.10	7.68	
T14	1.87	-0.02	1.87	-0.02	7.58	5.53
A15	-	-	8.23	-0.07	8.45	6.55
G16	11.65	-0.17	11.57	**- 0.25**	8.16	6.23
G17	11.09	-0.08	11.05	- 0.12	7.70	
G18	10.85	-0.11	10.79	**- 0.17**	7.77	
T19	1.92	0.00	1.92	0.00	7.77	6.42
G20	11.15	-0.05	11.14	- 0.06	7.68	
G21	11.20	-0.08	11.18	- 0.10	7.78	6.00
G22	10.91	-0.07	10.88	**-** 0.10	7.51	
T23	1.60	+0.20	1.66	+0.25	7.15	5.75

[a]$\Delta\delta = \delta_{bound}$-$\delta_{free.}$ R = 1.0; [b] $\Delta\delta = \delta_{bound}$-$\delta_{free.}$ R = 2.0.

Table S3. Selected chemical shift values of the complex **1**/ Pu22T14T23.

	**H2/NH/Me**	***Δδ*[b]**
T4	1.64	-0.02
G7	11.60	**- 0.08**
G8	11.15	0.00
G9	10.75	**+ 0.22**
T10	1.95	0.00
G11	11.51	**- 0.12**
G12	11.36	**- 0.06**
G13	11.02	**+0.05**
T14	1.85	0.00
A15	8.45	0.00
G16	11.70	**- 0.12**
G17	11.15	**- 0.04**
G18	10.88	**- 0.08**
T19	1.95	0.00
G20	11.26	**+ 0.05**
G21	11.28	+0.01
G22	11.00	+0.02
T23	1.55	+0.15

[a]$\Delta\delta = \delta_{bound}-\delta_{free.}$ R = 1.0; [b] $\Delta\delta = \delta_{bound}-\delta_{free.}$ R = 2.0.

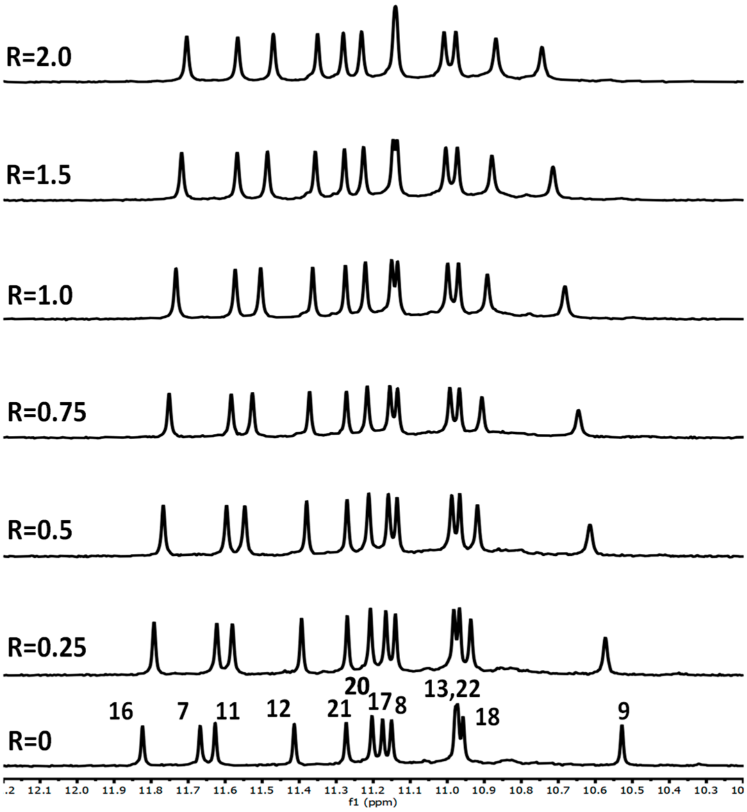

Figure S1. Imino proton region of the 1D NMR titration spectra of Pu22 with **2** at 25 °C in H_2O/D_2O (9:1), 25 mM KH_2PO_4, 70 mM KCl, pH 6.9, at different R = [**2**]/[DNA] ratios.

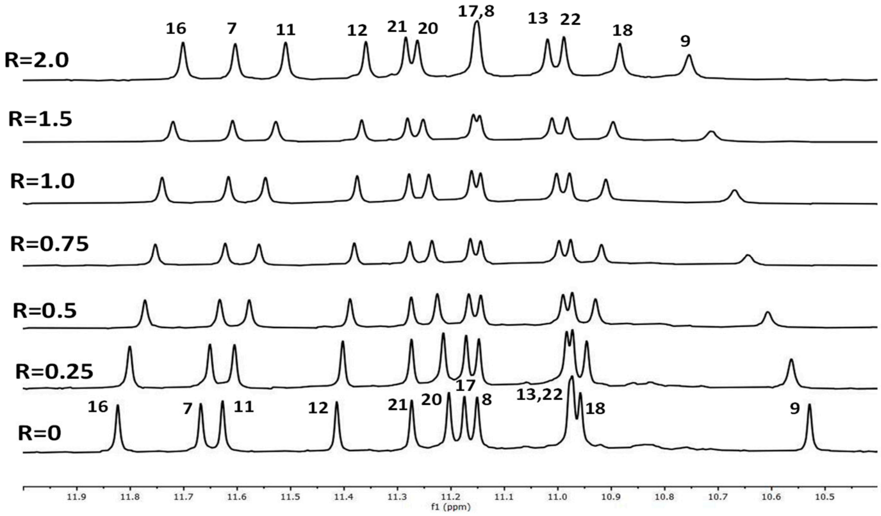

Figure S2. Imino proton region of the 1D NMR titration spectra of Pu22 with **1** at 25 °C in H_2O/D_2O (9:1), 25 mM KH_2PO_4, 70 mM KCl, pH 6.9, at different R = [**1**]/[DNA] ratios.

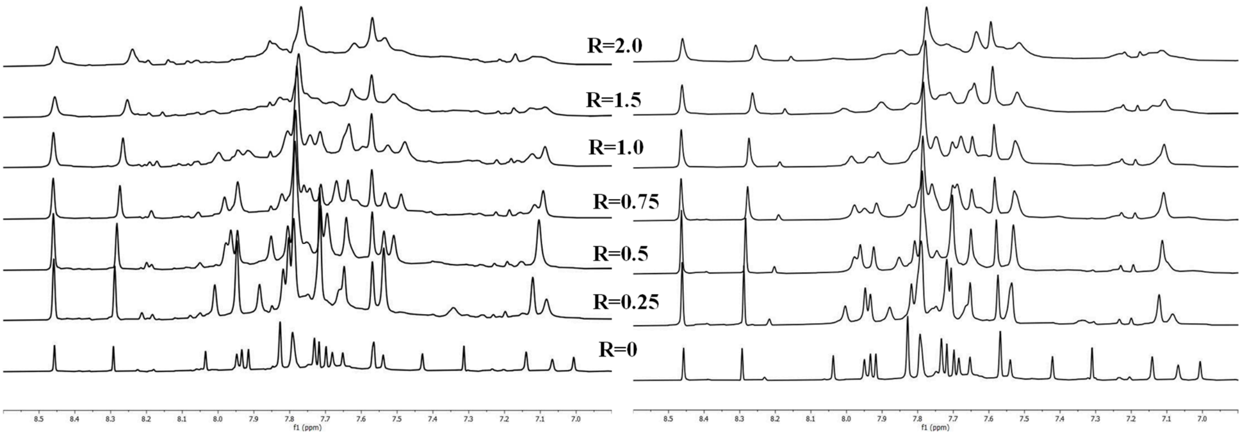

Figure S3. Aromatic protons region of the 1D NMR titration spectra of Pu22 with **4** (left) and **3** (right) at 25 °C in H_2O/D_2O (9:1), 25 mM KH_2PO_4, 70 mM KCl, pH 6.9, at different R = [ligand]/[DNA] ratios.

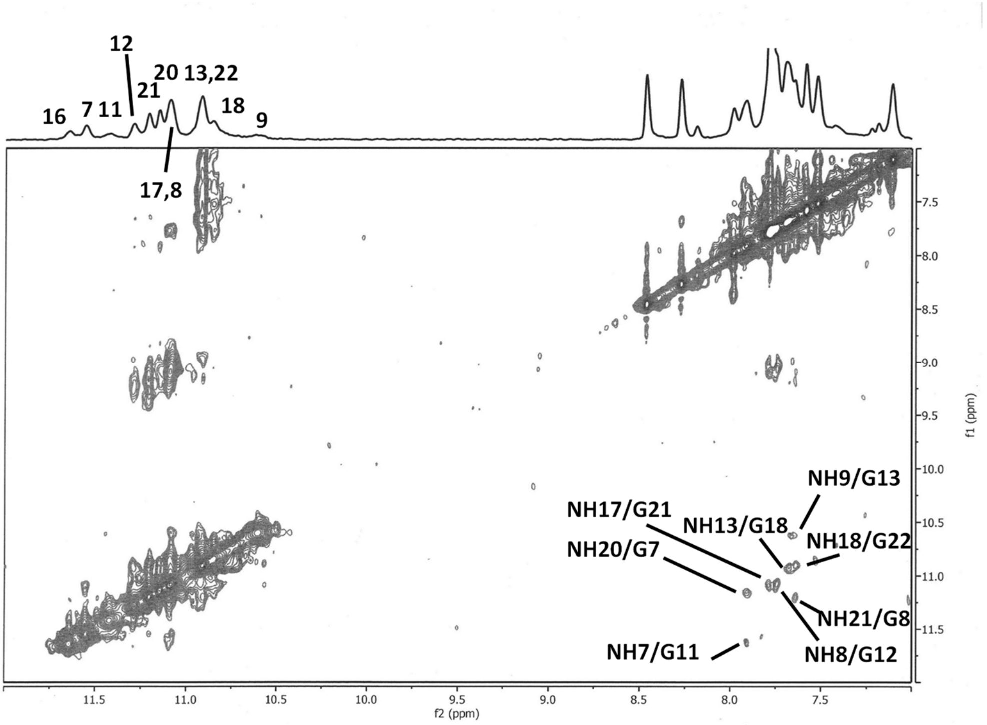

Figure S4. Selected region of the 2D-NOESY spectrum acquired at 25 °C in H_2O/D_2O (9:1), 25 mM KH_2PO_4, 70 mM KCl, pH 6.9, R= [**3**] / [DNA] = 1.0. G11NH–G16H8 and G16NH–G20H8 NOE cross-peaks were detected only at a ratio of 2.0.

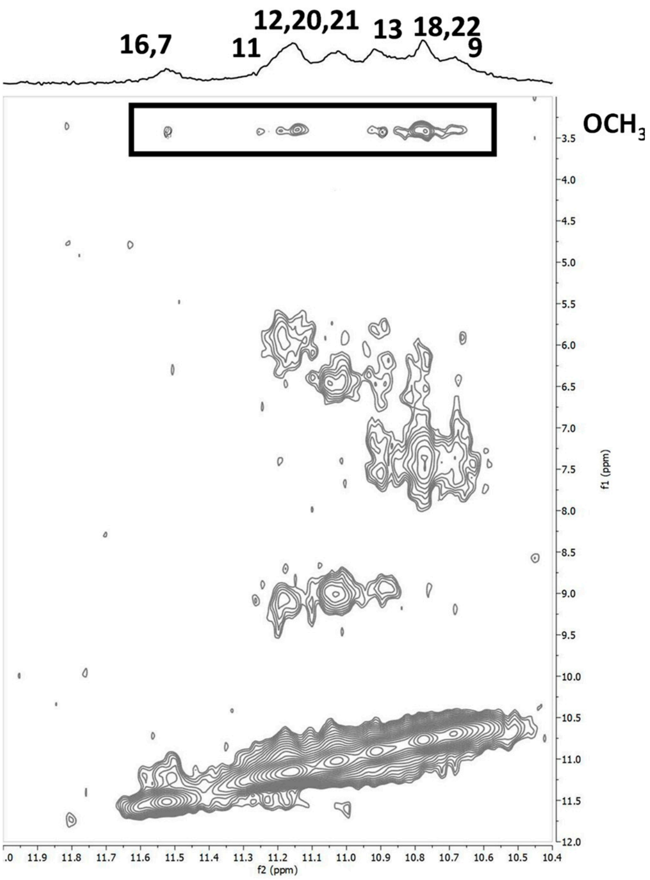

Figure S5. Imino proton region of the 2D-NOESY spectrum at 25 °C in H_2O/D_2O (9:1), 25 mM KH_2PO_4, 70 mM KCl, pH 6.9, at R= [**4**]/[DNA] =2.0. In the box the interactions between imino protons of PuT14T23 and OCH_3 of **4.**

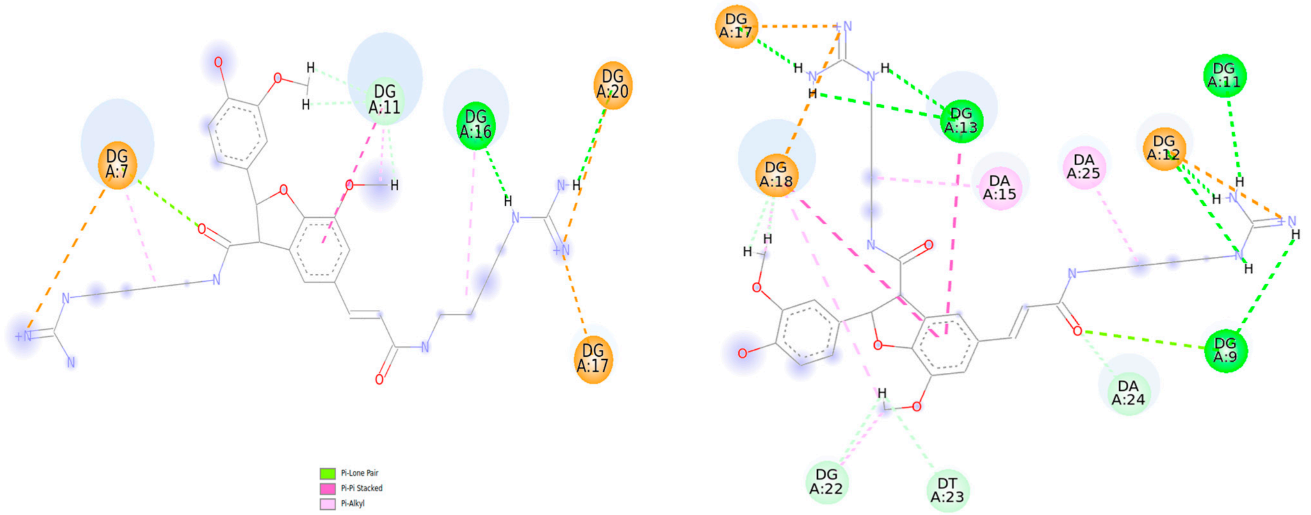

Figure S6. Two-dimensional representation of the molecular interactions between **1** and the Pu22T14T23. On the left 5′-end and on the right 3′-end binging site. Here, the colors used refer to the different interactions, as indicated in the legend.

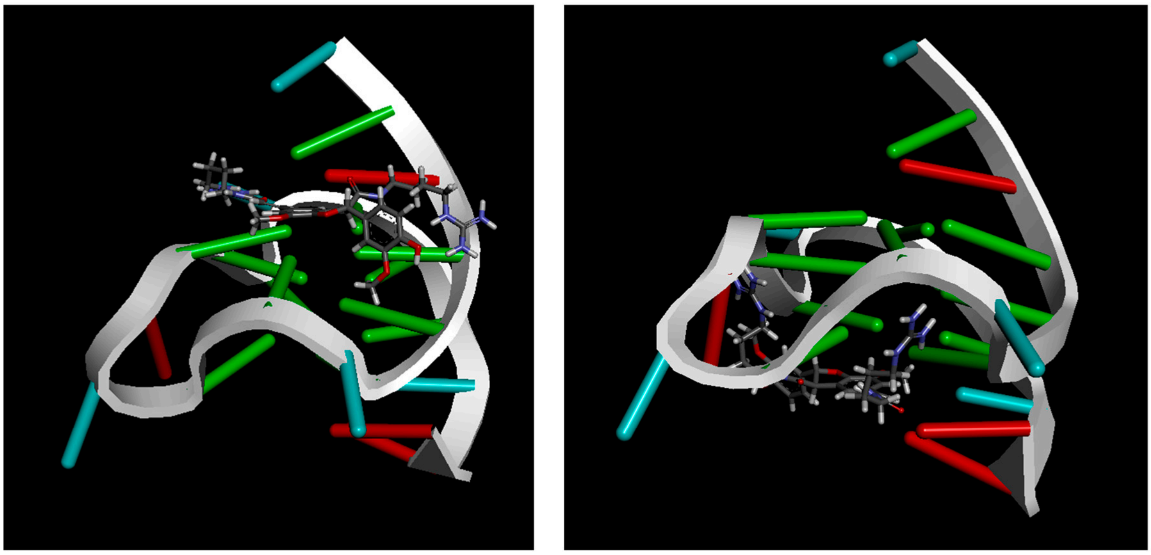

(a) (b)

Figure S7. Side viewsof the **1** complex with the c-MYC G-quadruplex sequence at the 5′-end (a) and 3′-end (b). The nucleotides are shown as: adenine in red, guanine in green, and thymine in blue.

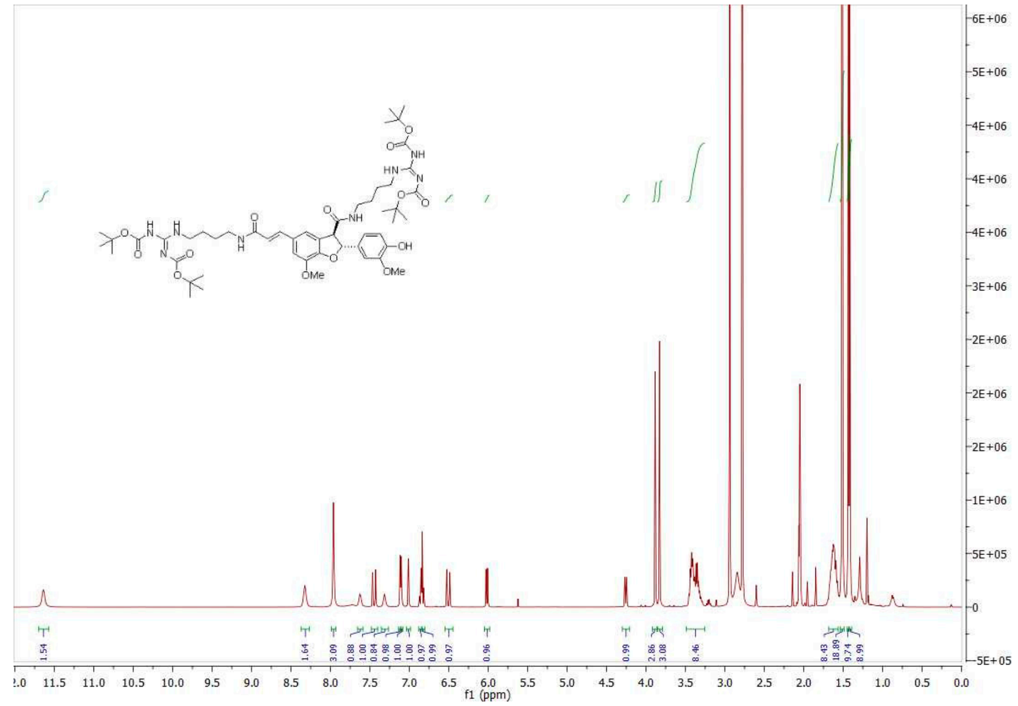

Figure S8. ^{1}H NMR spectrum of compound **11a**.

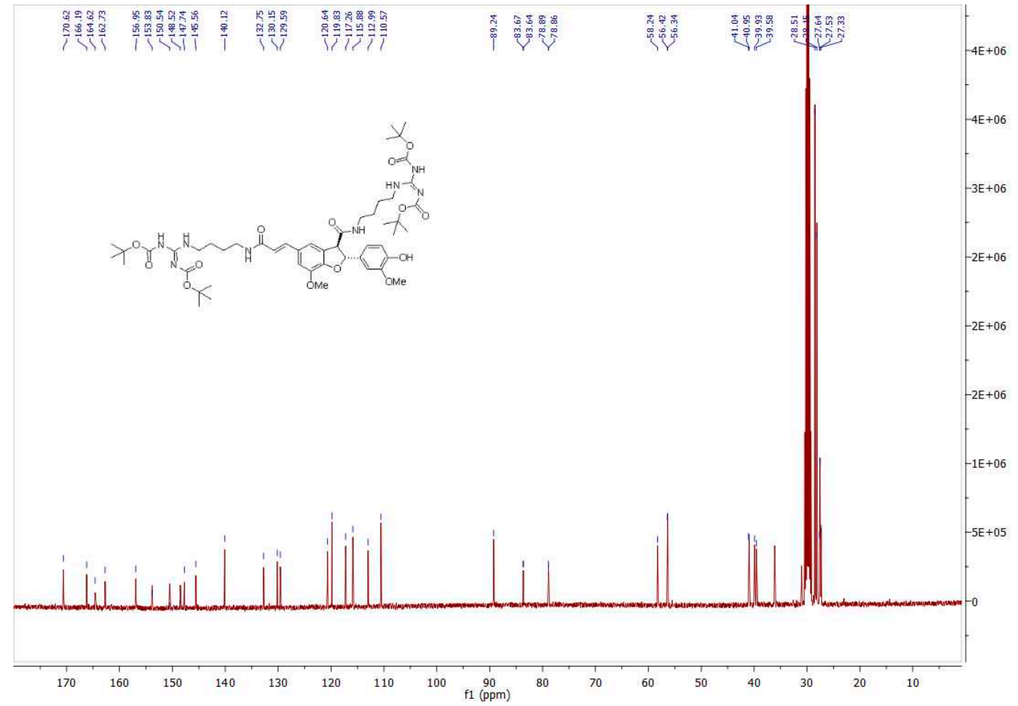

Figure S9. ^{13}C NMR spectrum of compound **11a**.

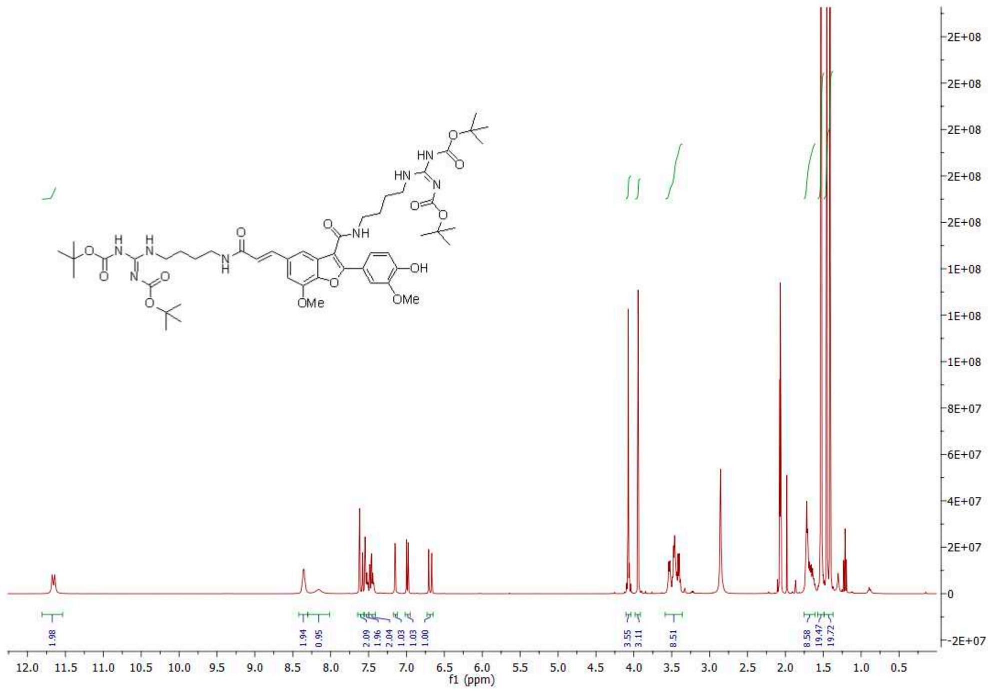

Figure S10. ^{1}H NMR spectrum of compound **12a**.

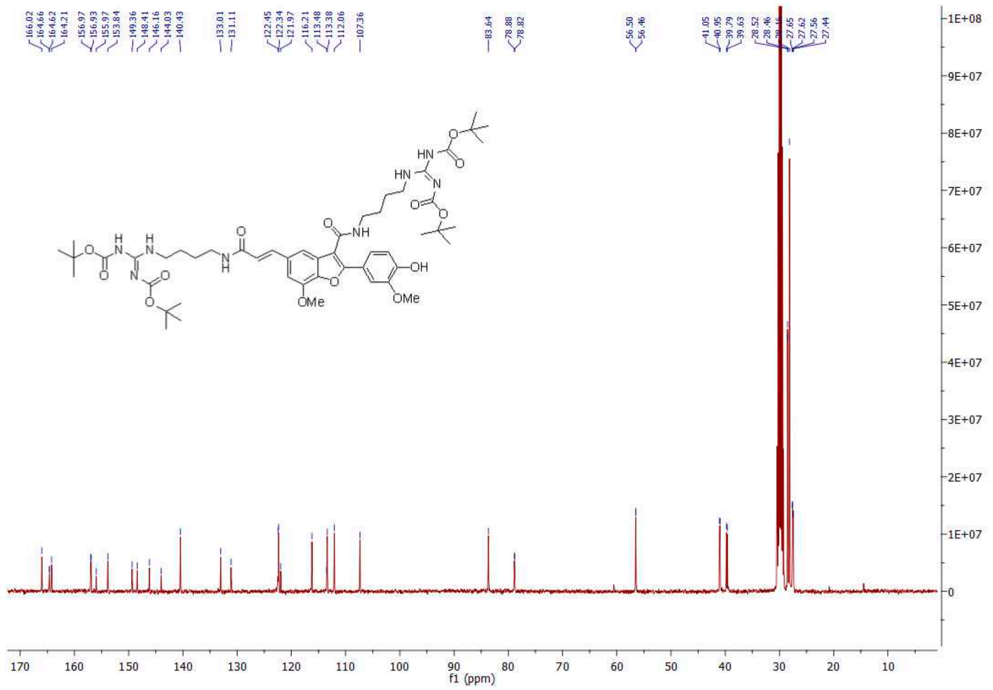

Figure S11. ^{13}C NMR spectrum of compound **12a**.

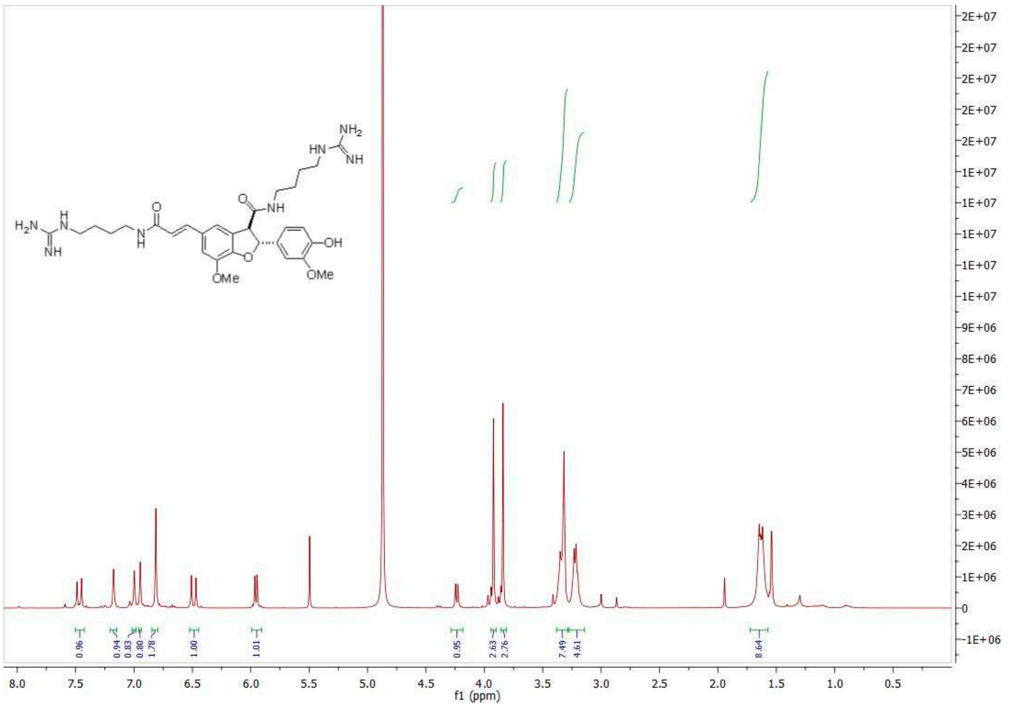

Figure S12. ^{1}H NMR spectrum of compound **1**.

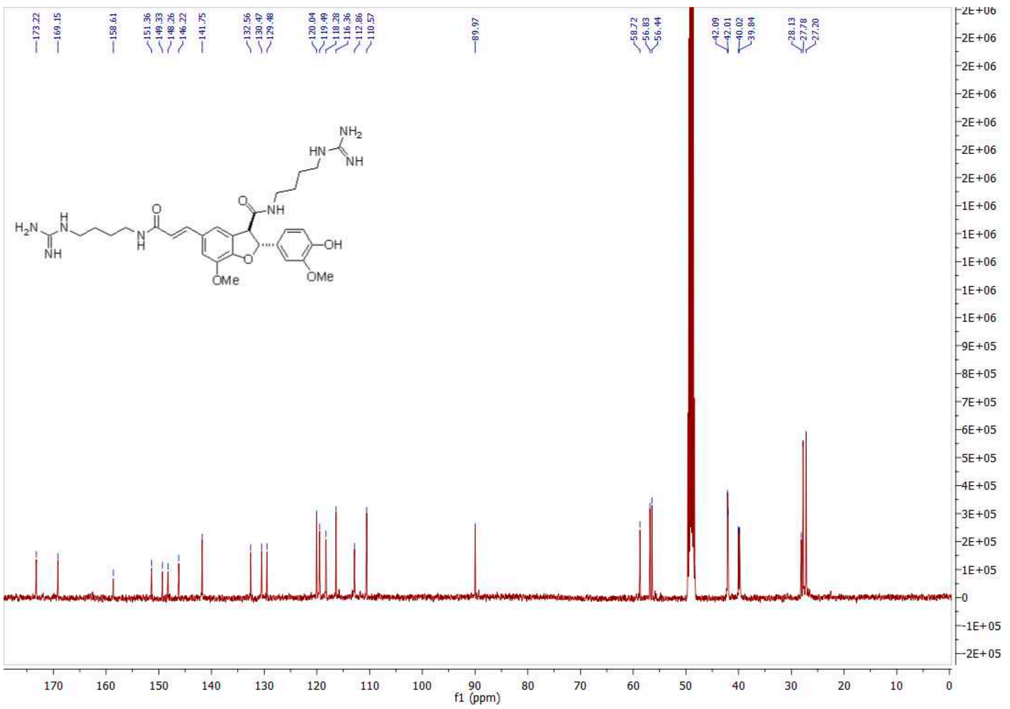

Figure S13. ^{13}C NMR spectrum of compound **1**.

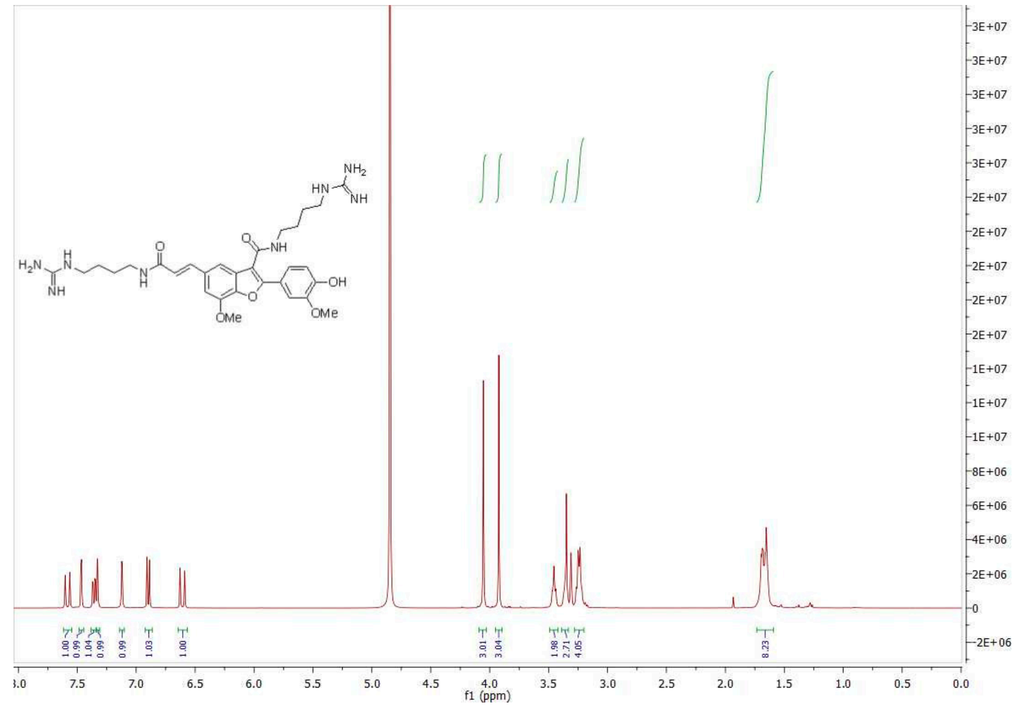

Figure S14. ^{1}H NMR spectrum of compound **3**.

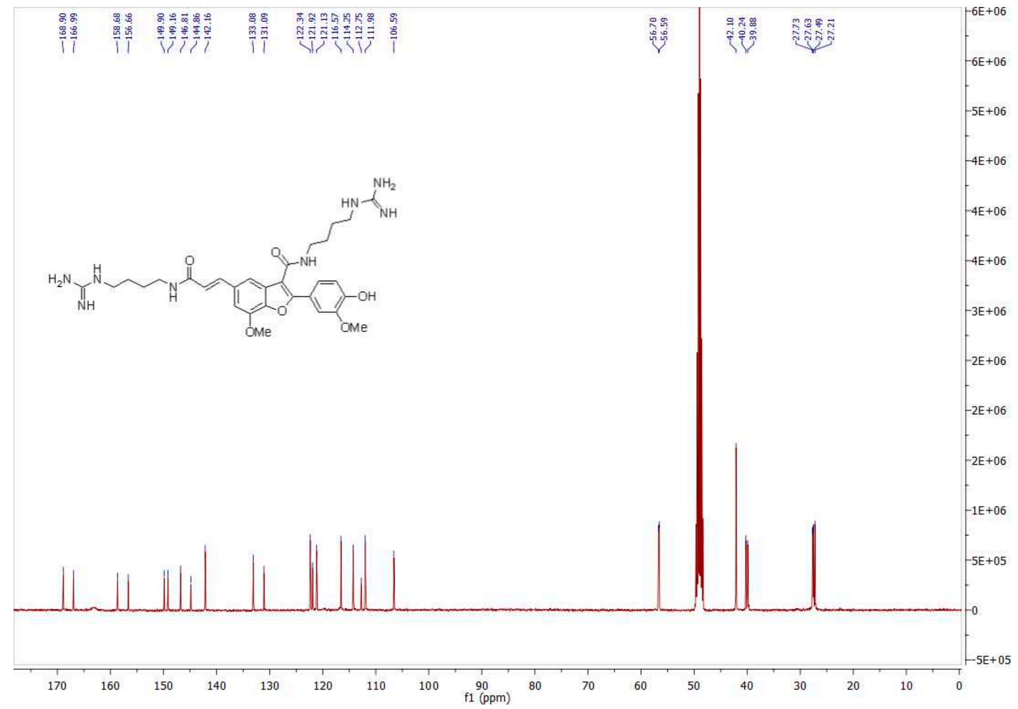

Figure S15. ^{13}C NMR spectrum of compound **3**.

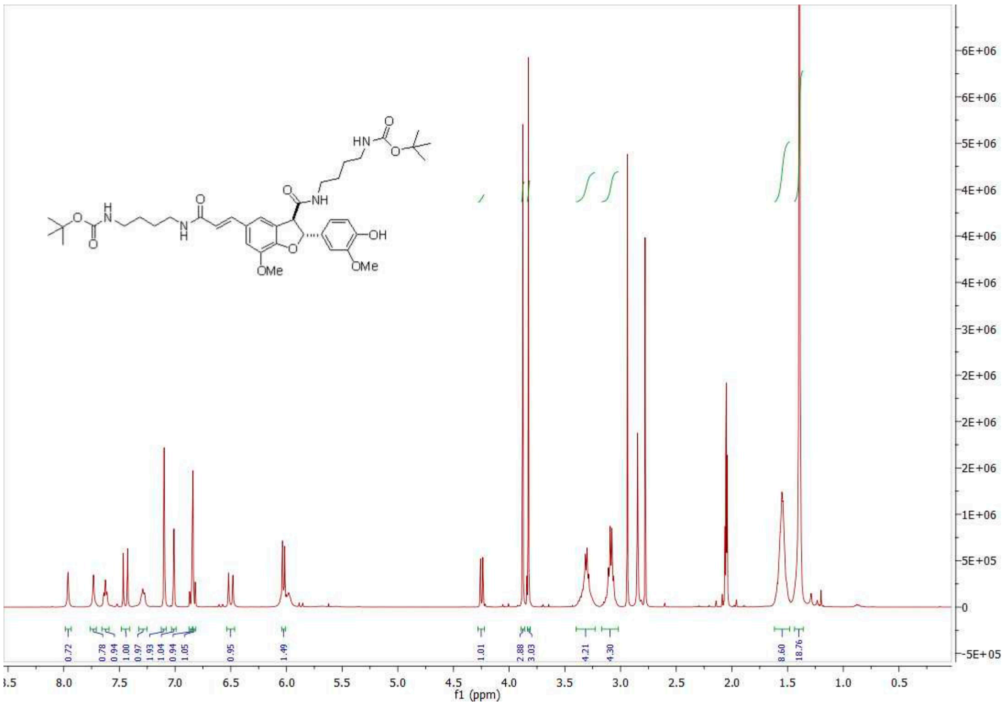

Figure S16. ^{1}H NMR spectrum of compound **11b**.

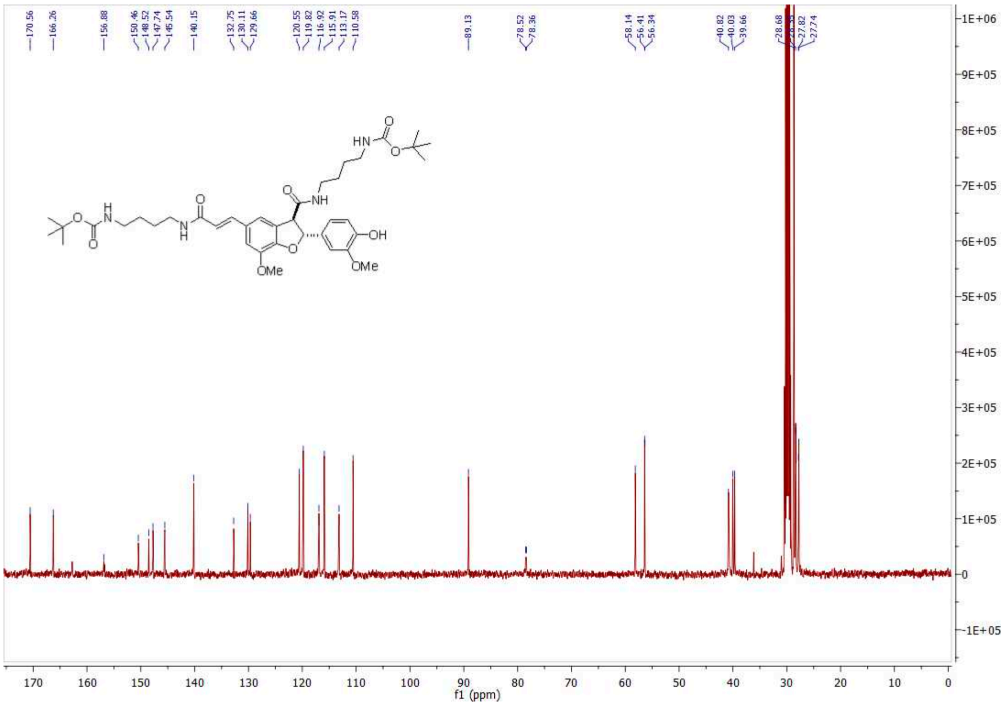

Figure S17. ^{13}C NMR spectrum of compound **11b**.

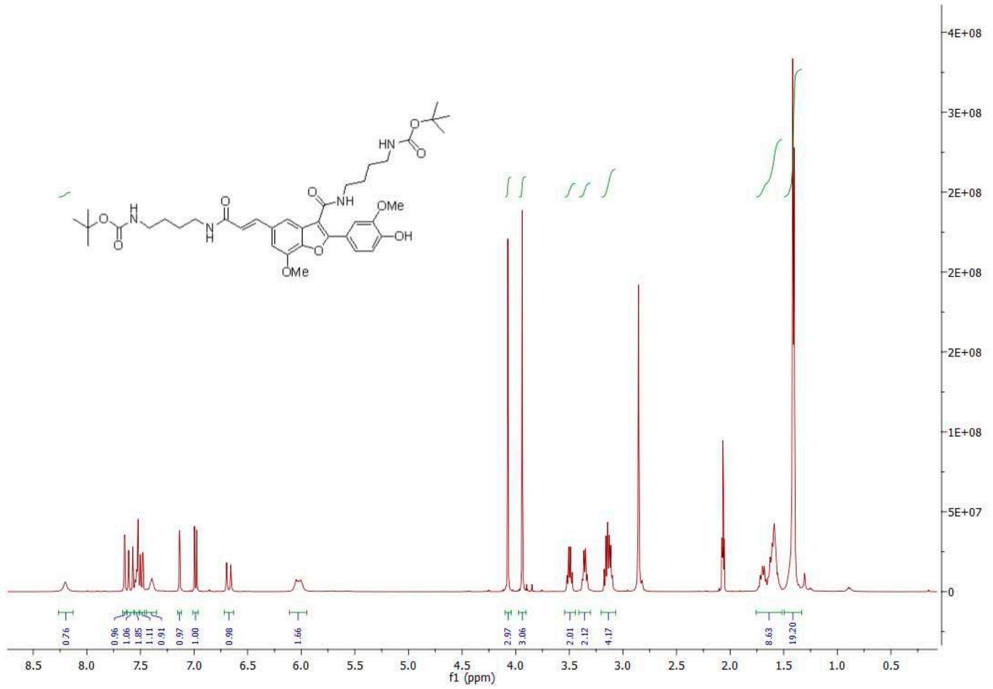

Figure S18. ^{1}H NMR spectrum of compound **12b**.

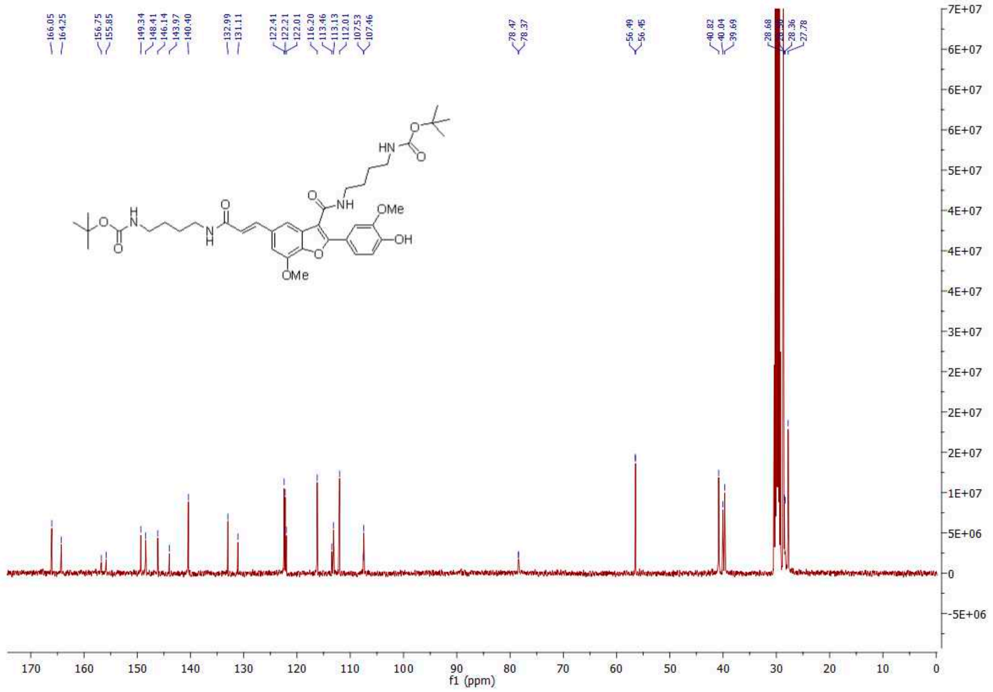

Figure S19. ^{13}C NMR spectrum of compound **12b**.

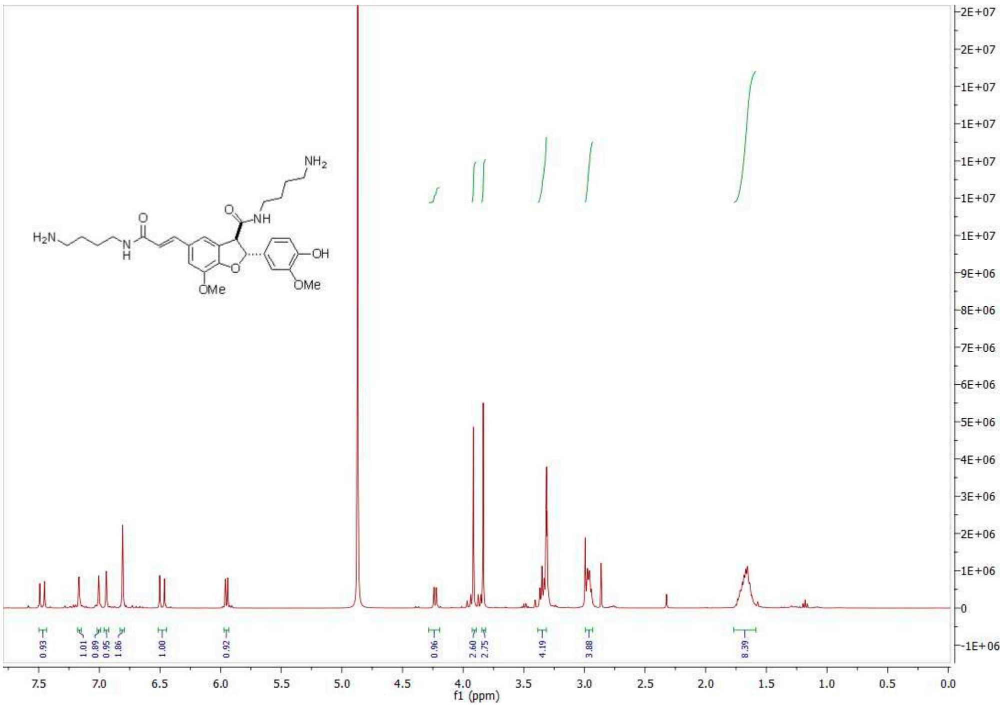

Figure S20. ^{1}H NMR spectrum of compound **2**.

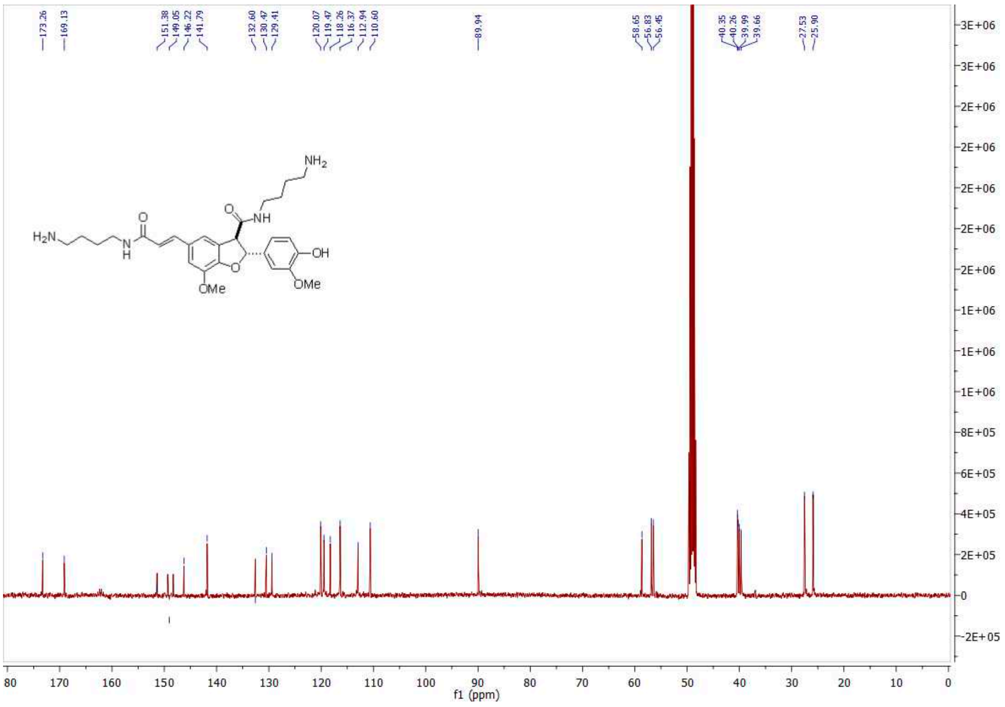

Figure S21. ^{1}H NMR spectrum of compound **2**.

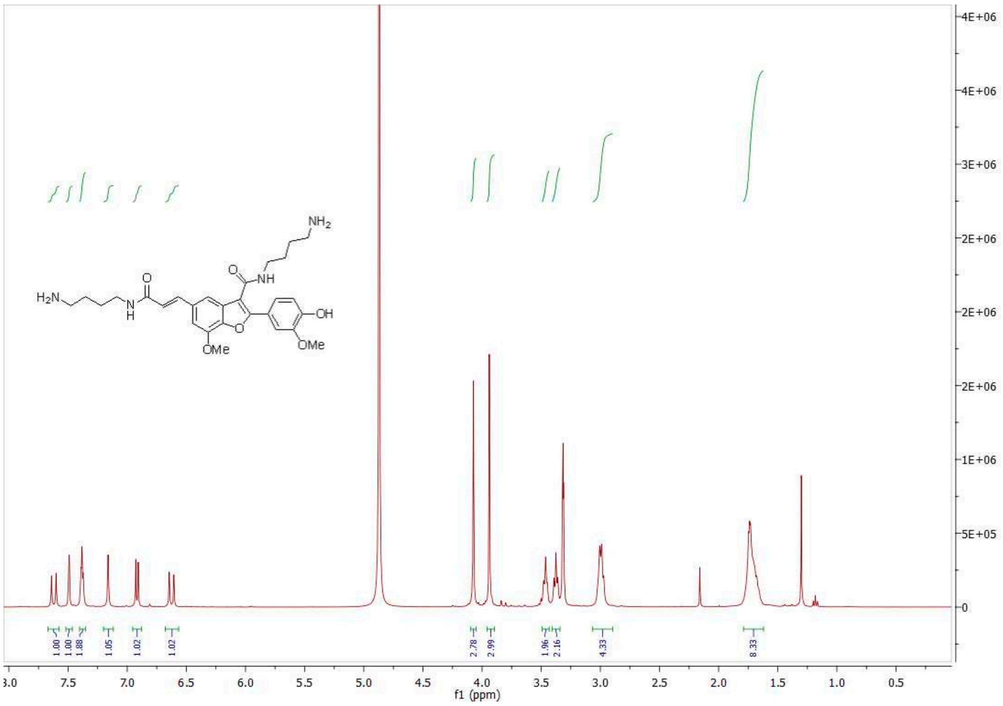

Figure S22. ^{1}H NMR spectrum of compound **4**.

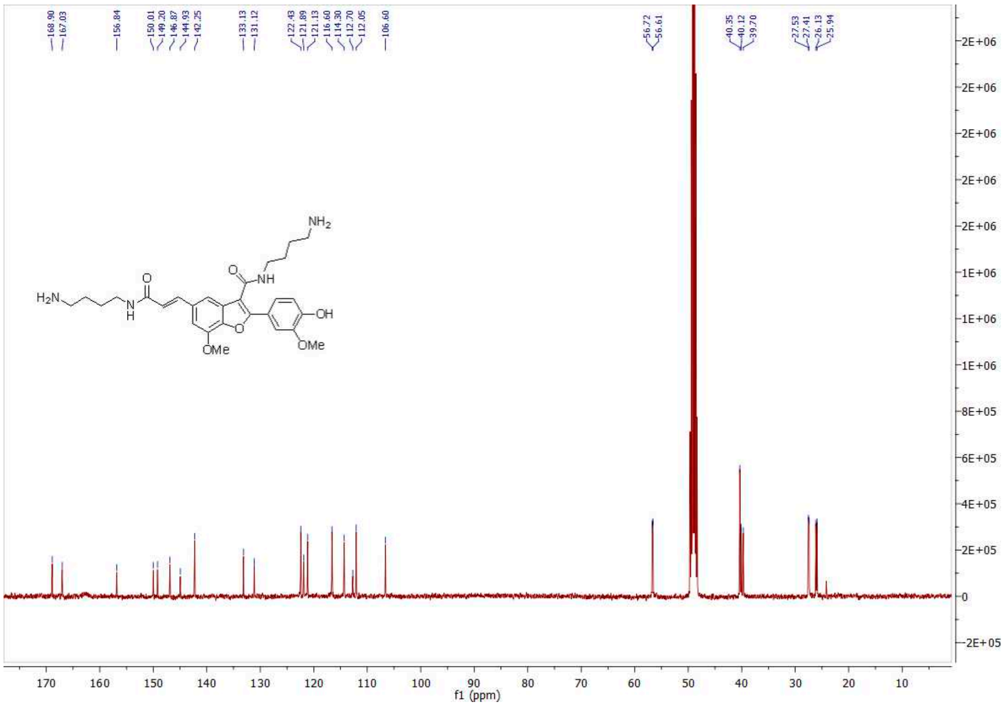

Figure S23. ^{1}H NMR spectrum of compound **4**.
